# Distinct phenotype and function of circulating Vδ1^+^ and Vδ2^+^ γδT-cells in acute and chronic hepatitis B

**DOI:** 10.1371/journal.ppat.1007715

**Published:** 2019-04-18

**Authors:** Kyong-Mi Chang, Daniel Traum, Jang-June Park, Suzanne Ho, Keisuke Ojiro, David K. Wong, Abdus S. Wahed, Norah A. Terrault, Mandana Khalili, Richard K. Sterling, Harry L. A. Janssen, Margaret C. Shuhart, Daryl T. Lau, Lewis R. Roberts, Geoffrey S. Johnson, David E. Kaplan, Michael R. Betts, William M. Lee, Anna S. F. Lok, for the Hepatitis B Research Network (HBRN)

**Affiliations:** 1 Medical Research, The Corporal Michael J. Crescenz VA Medical Center, Philadelphia PA, United States of America; 2 Department of Medicine, University of Pennsylvania Perelman School of Medicine, Philadelphia PA, United States of America; 3 Toronto Centre for Liver Disease, University of Toronto, Toronto, Ontario, Canada; 4 University of Pittsburgh Graduate School of Public Health, Pittsburgh PA, United States of America; 5 Department of Medicine, University of California, San Francisco, San Francisco CA, United States of America; 6 Department of Internal Medicine, Virginia Commonwealth University, Richmond VA, United States of America; 7 Harborview Medical Center, University of Washington Medical Center, Seattle WA, United States of America; 8 Department of Medicine, Beth Israel Deaconess Medical Center, Boston MA, United States of America; 9 Department of Internal Medicine, Mayo Clinic, Rochester MN, United States of America; 10 Department of Internal Medicine, University of Texas Southwestern Medical Center, Dallas TX, United States of America; 11 Department of Internal Medicine, University of Michigan, Ann Arbor MI, United States of America; Nationwide Children's Hospital, UNITED STATES

## Abstract

Hepatitis B virus (HBV) persists with global and virus-specific T-cell dysfunction, without T-cell based correlates of outcomes. To determine if γδT-cells are altered in HBV infection relative to clinical status, we examined the frequency, phenotype and function of peripheral blood Vδ1^+^ and Vδ2^+^γδT-cells by multi-parameter cytometry in a clinically diverse North American cohort of chronic hepatitis B (CHB), acute hepatitis B (AHB) and uninfected control subjects. We show that circulating γδT-cells were comprised predominantly of CD3^hi^CD4^-^ Vδ2^+^γδT-cells with frequencies that were 2–3 fold higher among Asian than non-Asian Americans and inversely correlated with age, but without differences between CHB, AHB and control subjects. However, compared to control subjects, CHB was associated with increased Tbet^hi^Eomes^dim^ phenotype in Vδ2^+^γδT-cells whereas AHB was associated with increased Tbet^hi^Eomes^dim^ phenotype in Vδ1^+^γδT-cells, with significant correlations between Tbet/Eomes expression in γδT-cells with their expression of NK and T-cell activation and regulatory markers. As for effector functions, IFNγ/TNF responses to phosphoantigens or PMA/Ionomycin in Vδ2^+^γδT-cells were weaker in AHB but preserved in CHB, without significant differences for Vδ1^+^γδT-cells. Furthermore, early IFNγ/TNF responses in Vδ2^+^ γδT-cells to brief PMA/Ionomycin stimulation correlated inversely with serum ALT but not HBV DNA. Accordingly, IFNγ/TNF responses in Vδ2^+^γδT-cells were weaker in patients with CHB with hepatitis flare compared to those without hepatitis flares, and this functional deficit persisted beyond clinical resolution of CHB flare. We conclude that circulating γδT-cells show distinct activation and differentiatiation in acute and chronic HBV infection as part of lymphoid stress surveillance with potential role in clinical outcomes.

## Author summary

We examined circulating γδT-cells in a North American cohort with chronic hepatitis B (CHB) and acute hepatitis B (AHB) compared to uninfected control subjects. While frequencies and composition of circulating γδT-cells were preserved in AHB and CHB, γδT-cells showed distinct and innate phenotypes based on the expression of Tbet/Eomes in association with various NK/T-cell markers. Notably, IFNγ/TNF responses to phosphoantigens and PMA/Ionomycin were preserved in CHB, but weaker in AHB compared to uninfected control subjects, in association with NKG2A/CD94 but not PD1. Furthermore, early IFNγ/TNF responses in Vδ2^+^ γδT-cells to brief PMA/Ionomycin stimulation showed significant inverse correlations with serum alanine aminotransferase, a measure of hepatocellular injury, and were persistently deficient in CHB subjects with hepatitis flare compared to those without such flares. Finally, Vδ2^+^ γδT-cells were significantly enriched for Tbet^hi^Eomes^dim^ phenotype in associations with their expression of NK and T-cell activation and regulatory markers, suggesting a role for Tbet in γδT-cell differentiation and function. We conclude that circulating γδT-cells show distinct activation and differentiation in acute and chronic HBV infection as part of lymphoid stress surveillance with potential role in clinical outcomes.

## Introduction

Hepatitis B virus (HBV) is an important human pathogen with a global impact in morbidity and mortality. As HBV is generally non-cytopathic, liver disease pathogenesis is largely immune-mediated with rapid progression to cirrhosis and cancer in some and minimal disease progression in others [1–3]. A critical role for conventional T-cells in viral clearance and liver disease has been shown in animal models and inferred in patients [4–9]. However, in patients with chronic hepatitis B (CHB), both HBV-specific and global T-cells are functionally suppressed due to continued antigenic stimulation, inflammation and the induction of multiple regulatory pathways [4–12]. Furthermore, there are no distinct T-cell based immune signatures for the dynamic clinical and virological phases of CHB [10]. These findings also raised the possiblity for alternate mechanisms beyond conventional T-cells in CHB pathogenesis.

In this context, γδT-cells are highly effector-like non-conventional CD3^+^ T-cells with T-cell receptors (TCR) comprised of γ and δ chains [13, 14]) and features of both adaptive and innate immune cells [15–18]. Among human γδT-cell subsets, Vδ2^+^ γδT-cells that co-express Vγ9 TCR with a CD3^hi^CD4^-^ phenotype are the major subset of circulating γδT-cells with frequencies of 2–4% in healthy adults, whereas Vδ1^+^ and Vδ3^+^ γδT-cells are detected in tissue compartments [15, 19, 20]. Unlike conventional αβT-cells, γδT-cells can be activated through MHC dependent and independent manners via multiple mechanisms including TCR, natural killer (NK) receptors and other receptors that can sense cellular stress and infection [15–18, 21–24]. In particular, Vδ2^+^ γδT-cells are activated by pyrophosphate molecules or “phosphoantigens” derived from the eukaryotic mevalonate pathway or microbial non-mevalonate pathway for isoprenoid synthesis [16, 25–27]. In humans, B7 family molecule butyrophilin subfamily 3, member A1 (BTN3A1) plays a key role in activating Vδ2^+^ γδT-cells by binding phosphoantigens that accumulate in stressed, transformed or infected cells [16, 25–28]. Furthermore, γδT-cells participate in lymphoid stress surveillance by sensing cellular stress associated with infection, inflammation and transformation [29, 30]. Because γδT-cells can be rapidly activated to lyse infected or malignant cells and to produce effector cytokines such as IFNγ and TNF or IL17 [15–18], there are ongoing efforts to harness γδT-cells in immunotherapy against cancer and viral infections [31–35].

Relevant for microbial pathogenesis, increased Vδ2^+^ γδT-cell frequency has been reported in infections with intracelluar pathogens such as mycobacterium tuberculosis, salmonella or malaria [17]. A protective role for γδT-cells in viral infections has been suggested by: increased Vδ2^+^ γδT-cells in elite HIV controllers [36], reduced Vδ2^+^ γδT-cell frequency associated with poor HIV-related outcomes [37–39], and Vδ1^+^ γδT-cell expansion associated with the resolution of cytomegalovirus (CMV) infection [40–42]. A role for γδT-cells in HBV immunobiology was first suggested in experimentally HBV-infected chimpanzees with hepatic induction of γδTCR-associated genes leading to viral clearance [43]. Findings in patients with CHB have been more conflicting [44–48], with reduced Vδ2^+^ γδT-cell frequencies in one study [44] but not another [48]. Both pathogenic and regulatory roles have been suggested by enhanced cytolytic potential reported in patients with HBV-associated acute-on-chronic liver failure [46] and CD8 T-cell exhaustion by inducing myeloid-derived suppressor cells in a mouse model [49].

Here, we compared the frequency, phenotype and function of circulating γδT-cells in human subjects with CHB relative to those with acute hepatitis B (AHB) and uninfected normal control subjects (NC), initially hypothesizing that their frequency and/or function will be reduced in CHB with increased disease activity. Contrary to our hypothesis, we found that γδT-cells are preserved in circulating frequencies in CHB and AHB, with distinct innate-like phenotype and/or effector function compared to uninfected control subjects. In particular, compared to uninfected controls, AHB subjects showed weaker IFNγ/TNF responses in Vδ2^+^ γδT-cells that improved with the resolution of AHB whereas CHB subjects showed with preserved IFNγ/TNF responses in Vδ2^+^ γδT-cells. Furthermore, in CHB, IFNγ/TNF responses to brief PMA/Ionomycin stimulation in Vδ2^+^ γδT-cells correlated inversely with serum alanine aminotransferase (ALT), with persistent deficit in patients with hepatitis ALT flares compared to those without flares. These findings provide new insights to γδT-cells with relevance to HBV pathogenesis.

## Methods

### Study subjects

Adults with CHB and acute hepatitis B (AHB) were enrolled into the Hepatitis B Research Network (HBRN) Adult Cohort Study sponsored by the National Institute for Diabetes and Digestive and Kidney Diseases (NIDDK), as previously described [50]. Subjects with hepatic decompensation, liver cancer, liver transplant, current hepatitis B antiviral therapy, known HIV co-infection and inability or unwillingness to attend follow-up visits were excluded. A subset of HBRN participants (215 CHB, 12 AHB) were recruited into the ancillary Immunology Study with additional informed consent for immunology blood draws from the following clinical centers: Toronto (University of Toronto), Dallas (University of Texas Southwestern), San Francisco (University of California San Francisco, California Pacific Medical Center); Richmond (Virginia Commonwealth University); Seattle (Virginia Mason Medical Center and University of Washington); Minnesota (University of Minnesota and Mayo Clinic), Boston (Beth Israel Deaconness Medical Center, Massachusetts General Hospital) and Chapel Hill (University of North Carolina), as previously described [10]. Among 215 CHB subjects initially enrolled into the Immunology Study, we included 189 in this study, excluding 10 who were found to be on antiviral therapy at the time of immunology blood draw and 16 without concurrent serum alanine aminotransferase (ALT) level.

Thirty-four HBsAg-negative normal control subjects (NC) were recruited from the HBRN Immunology Center in Philadelphia (University of Pennsylvania and Corporal Michael J. Crescenz Veterans Affairs Medical Center), Toronto and Dallas, including 29 with a history of prior HBV vaccination. NC subjects were recruited based on the absence of known liver disease, autoimmune disease, immunosuppression and active medical conditions that preclude large volume research blood draws. They were also negative for serum HBsAg, antibody to hepatitis C virus (anti-HCV) and antibody to human immunodeficiency virus (anti-HIV).

As shown in **Table 1A,** CHB, NC and AHB groups did not differ significantly in age or sex distribution, although the CHB group showed a marked Asian predominance consistent with the overall HBRN adult cohort [10, 50]. Asians with CHB showed higher HBV DNA levels with higher prevalence of positive hepatitis B e antigen (HBeAg) status and infection with HBV genotypes B or C, compared to Non-Asians with CHB (**Table 1B**). As for liver biopsy (**Table 1B**, bottom), 50/189 (26%) CHB subjects underwent liver biopsy within two years of enrollment, with histological cirrhosis in 3/50 subjects (6%).

**Table 1:** Patient characteristics.

**A. Demographic characteristics of CHB, NC and AHB groups**				
	**CHB (n = 189)**	**NC (n = 34)**	**AHB (n = 12)**	CHB vs NC vs AHB*
Median age	39	44	41	0.14
(min, max)	(18, 76)	(22, 72)	(24, 54)	
% Males (n)	54%	41%	75%	0.12
% Asians	84%	47%	0%	< .0001
%White	11%	44%	67%	
%Black	5%	9%	25%	
Mixed	1%	0%	8%	
**B. CHB participants: Comparison between Asians and Non-Asians**				
	**All (n = 189)**	**Asian (n = 158)**	**Non-Asian (n = 31)**	Asian vs NonAsian*
Median age	39	39	45	0.09
(min, max)	(18, 76)	(18, 76)	(18, 72)	
% Males (n)	54%	51%	71%	0.05
Median ALT/ULN	1.7	1.7	1	0.58
(min, max)	(0.5, 77.1)	(0.5, 34.5)	(0.5, 77.1)	
Median HBV DNA log IU/ml	4.9	5.3	3.9	0.04
(min, max)	(1, 9)	(1, 8.9)	(1.1, 9)	
%HBeAg+	47%	44%	23%	0.04
HBV genotype				
% Geno A	8%	3%	32%	< .0001
% Geno B	50%	59%	6%	
% Geno C	29%	34%	3%	
% Geno D	6%	2%	29%	
% Geno E	%	0%	16%	
# (%) with liver biopsy within 2 years of immune analysis	50/189 (26%)	42/158 (27%)	8/31 (26%)	
***#(%) cirrhosis on biopsy	3/50 (6%)	3/42 (7%)	0/8 (0%)	

*p-values by Kruskal Wallis (k = 3), Mann Whitney or Fisher's Exact test

***cirrhosis defined by Knodell and/or Metavir score 4. Among 36 subjects with more detailed analyses of γδT-cell phenotype and function, 8 had liver biopsy results with 1 with cirrhosis.

### Ethics statement

The study received approval by the institutional review board or equivalent committee(s) for each of the centers participating in patient recruitment as stated above and previously described [10]: Toronto (University of Toronto), Dallas (University of Texas Southwestern), San Francisco (University of California San Francisco, California Pacific Medical Center); Richmond (Virginia Commonwealth University); Seattle (Virginia Mason Medical Center and University of Washington); Minnesota (University of Minnesota and Mayo Clinic), Boston (Beth Israel Deaconness Medical Center, Massachusetts General Hospital) and Chapel Hill (University of North Carolina). All subjects were adults and provided written informed consent. The study was conducted according to the principles in the Declaration of Helsinki.

### Clinical grouping of HBV-infected subjects

Clinical phase of each HBV-infected subject was assigned based on medical history and laboratory results as previously described as immune tolerant (IT), HBeAg^+^ immune active (IA+), HBeAg^-^ immune active (IA-) or inactive carrier (IC) status [10]. ALT levels were normalized by the upper limits of normal (ULN) for ALT (30 U/L for men and 20 U/L for women) as ALT/ULN ratio. ALT flare was defined by ALT/ULN ≥10 (ALT 300 U/L for males, 200 U/L for females). Subjects identified with an ALT flare were asked to return for additional immunology blood draw within 1–4 weeks of meeting criteria for ALT flare and again within 12–24 weeks from initial hepatitis flare or before starting antiviral therapy if not resolved. Acute hepatitis B (AHB) was defined by acute ALT elevation in the presence of HBsAg and IgM antibody to hepatitis B core antigen (IgM anti-HBc) without a previous history of HBsAg positivity. The Immunology Center personnel were blinded to clinical parameters while conducting immune assays.

### Peripheral blood mononuclear cells (PBMC)

PBMC were isolated from blood drawn in lavender-top plasma tubes (ethylenediaminetetraacetic acid or EDTA as additive) using Ficoll-Histopaque (Sigma Chemical Co., St Louis, MO) density centrifugation and resuspended in complete media with 10% human male AB serum as described [10]. PBMC isolation was performed within 24 hours of blood draw.

### Antibodies and reagents

Fluorescent monoclonal antibodies (mAbs) were purchased as follows: anti-CD8, anti-CD56, anti-CD94, anti-NKG2D, anti-CTLA-4 and anti-TNF from BD Bioscience (San Jose, CA); anti-CD127, anti-CD3, anti-CD28 and anti-Eomesodermin (Eomes) from eBioscience (San Diego, CA); anti-PD-1, anti-Tbet and anti-IFNγ from BioLegend (San Diego, CA); anti-NKG2A from R&D Systems (Minneapolis, MN); anti-Vδ1 TCR (clone REA173) from Miltenyi Biotec (San Diego, CA); anti-Vδ2 TCR (clone B6), anti-Vγ9 TCR (clone B3) and pan-γδ TCR (clone B1) from BioLegend (San Diego, CA). Dead cells were excluded using Aqua dead cell stain kit (Life Technologies). The phycoerythrin- or allophycocyanin-labeled CD1d tetramers were kindly provided by the NIH Tetramer Facility at Emory University (Atlanta, GA).

### Immunophenotyping

Cells were stained with fluorescent antibodies according to the manufacturer's instructions, acquired by FACSCanto (BD Biosciences, San Jose, CA) and analyzed with FlowJo (Tree Star Inc., San Carlos, CA). Magnetic beads coated with anti-mouse antibodies were used for compensation calculation (BD Biosciences, San Jose, CA) as described previously [51, 52].

CD3^hi^CD4^-^ T-cell frequency and phenotype in 189 CHB (including 39 with hepatitis flare), 34 NC and 12 AHB participants were examined by staining freshly isolated PBMC with a multi-parameter screening T-cell panel that included fluorescent antibodies for CD3, CD4, CD8, PD-1, CTLA-4, CD28 and CD127 [10]. More detailed γδT-cell analyses were performed with secondary FACS panels in 70 subjects with available cryopreserved PBMC (36 CHB, 27 NC and 7 AHB participants) with the inclusion of CD16, CD56, CD161, NKG2D, NKG2A, CD158a and/or CD94. Among the 36 CHB subjects, 14 with ALT flares (CHB-F) were first examined within 1–4 weeks of ALT flare. PBMCs from a second time point (at least 2 months from the initial time point) were also analyzed in 7 CHB-F and in 7 AHB subjects. Examination for γδTCR subtype (Vδ1, Vδ2, Vγ9), phenotype (T/NK markers), effector molecules (perforin, granzyme B) and transcription factor expression (Tbet, Eomes) was conducted directly ex-vivo. In select cases with available lymphocytes (11 CHB, 7 NC), PBMC were stained with metal-conjugated antibodies and acquired by CyTOF II as described [53, 54], for markers including perforin, granzyme, PD1, CD38, HLA DR, Ki67, TIGIT and Tim3. As Vδ2^+^ γδT-cells (but not Vδ1^+^ γδT-cells) co-expressed Vγ9 TCR with a distinct CD3^hi^CD4^-^ phenotype, Vδ2^+^ γδT-cells were defined by the expression of Vδ2 or Vγ9 TCR as well as CD3^hi^CD4^-^ phenotype based on available antibodies in our FACS panels.

### Analyses of γδT-cell effector functions

Effector functions in T-cell subsets (Vδ1^+^, Vδ2^+^, CD3^hi^CD4^-^, CD3^int^CD4^-^ and total CD3^+^ T-cells) were first examined in 36 CHB, 24 NC and 7 AHB subjects with available lymphocytes by intracellular cytokine staining in PBMC stimulated for 5 hours with 10 ng/ml phorbol 12-myristate 13-acetate (PMA) and 200 ng/ml Ionomycin in-vitro as previously described [55–57]. CD3^hi^CD4^-^ phenotype and Vγ9 TCR expression were used to define Vδ2^+^ γδT-cells in these cytokine stainings.

In 23 CHB, 11 controls and 7 AHB subjects with available PBMC, IFNγ, TNFα and/or IL-17 expression in immune subsets were further examined after 23 hours of phosphoantigen (pAg) stimulation in modified protocol based on published literature [58] with 0.5 million PBMC/well stimulated for 23 hours with: 1) media with 50 U/ml recombinant interleukin-2 (rIL2); 2) 20 microM zoledronic acid (Zol) (Sigma Aldrich) with 50 U/ml rIL2; 3) 1 nanoM (E)-4-hydroxy-3-methyl-but-2-enyl pyrophosphate (HMBPP) (Sigma Aldrich) with 50 U/ml rIL2; 4) 10 ng/ml PMA and 200 ng/ml Ionomycin. For 23 hour stimulation, 10 mcg/ml Brefeldin A (eBioscience) and 1X GolgiStop (BD Biosciences) were added after 16 hours to maximize intracellular signal [58]. In control assays comparing 23 and 8 hours of stimulation (**S1 Fig**), IFNγ/TNF responses in Vδ2^+^ γδT cells were greater with longer pAg stimulation (23 hours > 8 hours) as previously reported [58] whereas the opposite was seen for PMA/Ionomycin (8 hours > 23 hours). To avoid confusion between IFNγ/TNF responses to 5 versus 23 hours of stimulation, we referred to 5 hours of PMA/Ionomycin stimulation as being “brief” and providing “early” IFNγ/TNF response, while using “late” responses to refer to results from 23 hours of pAg or PMA/Ionomycin stimulation.

### Statistical analyses

Patient characteristics were compared using Pearson chi-square or Fisher’s exact test for discrete variables such as sex, race and genotype, and Kruskal Wallis test for continuous variables such as HBV DNA and ALT. Immune measures between distinct patient groups were compared using Mann-Whitney U (for two groups) and Kruskal-Wallis test (for more than two groups). When comparing 3 or more groups, first comparison was made with Kruskal Wallis, followed by further comparisons between 2 groups by Mann Whitney U if the initial Kruskal Wallis yielded p-value < 0.05. Correlated samples (e.g. immune measures within the same cells) were compared using matched pair signed-rank test. Correlation between two immune measures was assessed using non-parametric Spearman correlation and the corresponding test. Proportions across two groups were compared by Fisher's exact test and across more than two categories by chi-square test or exact chi-square tests as appropriate. P values below 0.05 were considered statistically significant.

## Results

### Circulating γδT-cells are comprised predominantly of CD3^hi^CD4^-^ Vδ2^+^ γδT-cells that are impacted in frequency by race/ethnicity and age but not HBV infection

**Fig 1:**
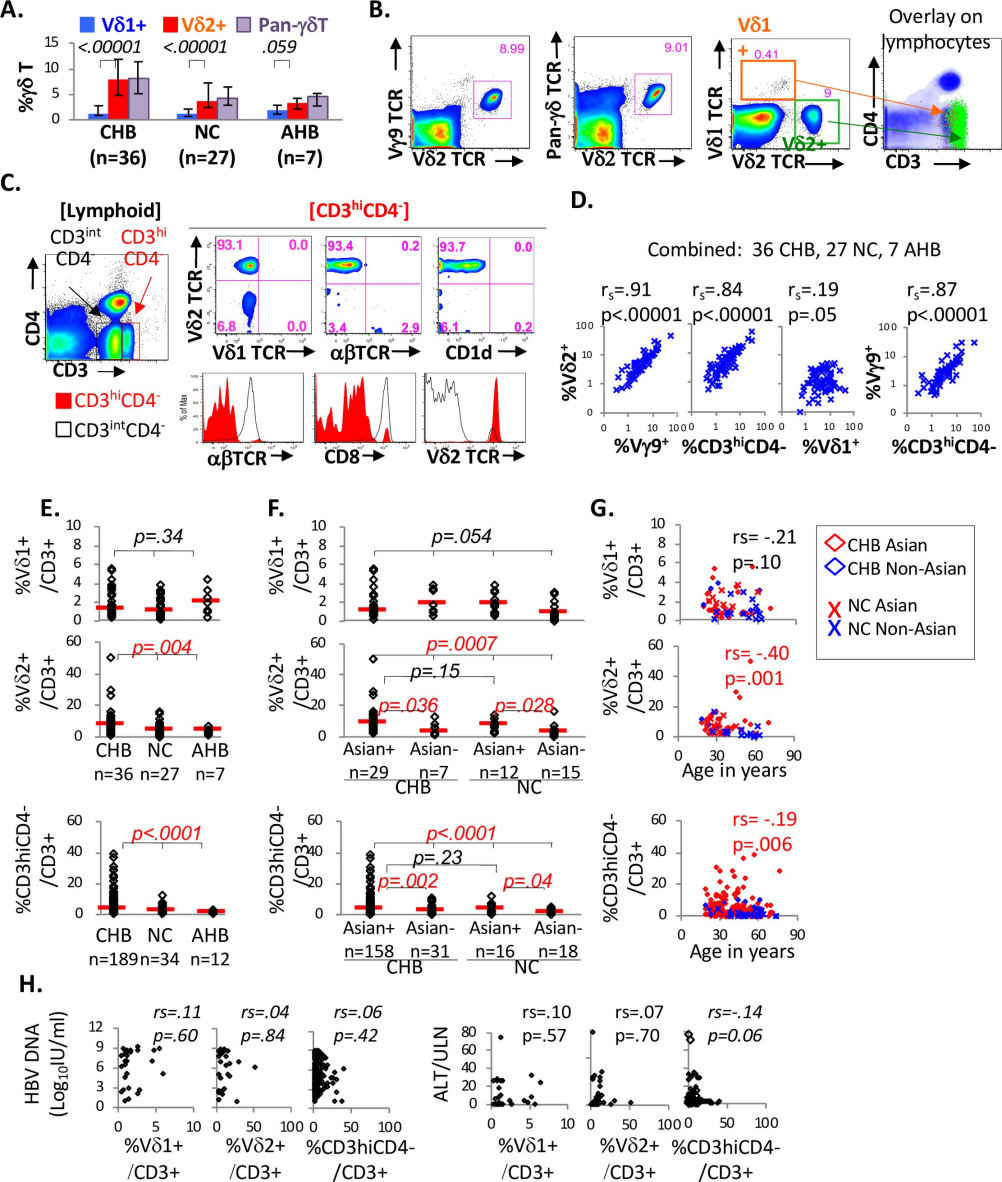
Circulating γδT-cells are comprised predominantly of CD3^hi^CD4^-^ Vδ2^+^ γδT-cells with frequencies that are impacted by host race/ethnicity and age but not HBV infection. **A. Comparison of circulating γδT-cell frequencies relative to HBV infection.** Bar graphs show median %**γδ**T-cells in CD3^**+**^ T-cells from 36 chronic hepatitis B (CHB), 27 uninfected normal controls (NC) and 7 acute hepatitis B (AHB) subjects determined by flow cytometry: %V**δ**1^**+**^ (blue bar), %V**δ**2^**+**^ (red bar) and %pan-**γδ**TCR^**+**^ (violet bar). Error bars indicate 25% and 75% interquartile ranges. Differences between %V**δ**1^**+**^ and %V**δ**2^**+**^ **γδ**T-cells were examined by non-parametric Mann-Whitney U. P-values below 0.05 were considered statistically significant. **B. Representative FACS appearance of γδT-cells.** Multi-color FACS analysis of peripheral blood mononuclear cells (gated on CD3^**+**^ viable singlet lymphocytes) show that V**δ**2^**+**^ cells co-express Vγ9 TCR and pan-**γδ**TCR but not V**δ**1 TCR. Overlay of V**δ**1^**+**^ (orange box gate) and V**δ**2^**+**^ cells (green box gate) onto lymphocytes (blue background) shows the CD3^hi^CD4^-^ phenotype of V**δ**2^**+**^ cells (green dots on the overlay) whereas Vδ1^**+**^ cells (orange dots on the overlay) show CD3^int^CD4^-^ appearance. **C. Enrichment of CD3**^**hi**^**CD4**^**-**^ **T-cells for Vδ2**^**+**^ **γδT-cells**. Gating strategy and distinct FACS appearance are shown for highly CD3-positive but CD4-negative cells (CD3^hi^CD4^-^) compared to CD3-intermediate CD4^-^ cells (CD3^int^CD4^-^). Upper panel shows pseudo-color plots of gated CD3^hi^CD4^-^ T-cells which are markedly enriched for Vδ2^+^ γδT-cells but not V**δ**1^**+**^ γδT-cells, αβTCR^+^ conventional T-cells or CD1d-reactive NKT-cells. Bottom panel shows histogram overlays for CD3^hi^CD4^-^ (red shade) and CD3^int^CD4^-^ (black line) T-cells with enrichment of CD3^int^CD4^-^ T-cells with αβTCR^+^ CD8^+^ T-cells and not Vδ2^+^ γδT-cells. **D. Correlations between circulating γδT-cell frequencies.** Significant correlations were detected between %V**δ**2^**+**^ **γδ**T-cells, %Vγ9^**+**^ **γδ**T-cells, and %CD3^hi^CD4^-^ T-cells, but not between %V**δ**1^**+**^ **γδ**T-cells and %V**δ**2^**+**^ **γδ**T-cells. **E. Comparison of γδT-cell frequencies in CD3**^**+**^ **T-cell compartment between CHB, NC and AHB groups**. Vδ1^+^ and Vδ2^+^ γδT-cell frequencies were compared between 36 CHB (29 Asians, 7 Non-Asians), 27 NC (12 Asians, 15 Non-Asians) and 7 AHB (7 Non-Asians) subjects with available cryopreserved PBMCs. CD3^hi^CD4^-^ γδT-cell frequencies were compared in 189 CHB (158 Asians, 31 Non-Asians), 34 NC (16 Asians, 18 Non-Asians) and 12 AHB (12 Non-Asians) subjects in freshly isolated PBMCs. Frequency differences between CHB, NC and AHB groups were calculated by non-parametric Kruskal Wallis (k = 3). P-values below 0.05 were considered significant and shown in red font. **F. Comparison of Vδ1**^**+**^**, Vδ2**^**+**^ **and CD3**^**hi**^**CD4**^**-**^ **γδT-cell frequencies between Asian and Non-Asian American subgroups with and without CHB**. Initial comparisons between 4 subgroups (Asian+ CHB, Asian- CHB, Asian+ NC, Asian- NC) were made by non-parametric Kruskal Wallis (k = 4), followed by further 2-group comparisons by Mann Whitney U if initial 4-way comparison showed p-values below 0.05. As shown, Vδ2^+^ and CD3^hi^CD4^-^ γδT-cell frequencies were greater in Asian Americans compared to Non-Asian Americans within CHB or NC groups, without significant differences between Asian American CHB versus Asian American NC subgroups. Horizontal red lines indicate median values. P-values below 0.05 were considered significant and shown in red font. **G. Inverse association between age and circulating Vδ2**^**+**^ **and CD3**^**hi**^**CD4**^**-**^ **γδT-cell frequencies.** Comparisons between age in years (x-axis) and %γδT-cells (y-axis) are shown for NC Asian Americans, (red X), NC Non-Asian Americans (blue X), CHB Asian Americans (red diamond) and CHB Non-Asian Americans (blue diamond), with significant inverse associations between age and %Vδ2^+^ or %CD3^hi^CD4^-^ γδT-cells but not Vδ1^+^ γδT-cells. Correlation coefficients and associated p-values for all subjects were determined by non-parametric Spearman rank order correlation test. **H. Lack of significant correlations between serum levels of HBV DNA or ALT and circulating γδT-cell frequencies.** Serum levels of HBV DNA or ALT on the y-axis are compared to circulating frequencies of Vδ1^+^, Vδ2^+^ and CD3^hi^CD4^-^ γδT-cells, with correlation coefficients and p-values by Spearman rank order correlation test.

We first examined the relative distribution of circulating γδT-cells in CHB, NC and AHB groups. As shown in **Fig 1A**, Vδ2^+^ γδT-cells showed significantly higher circulating frequencies than Vδ1^+^ γδT-cells in CHB (7.6% vs 1.1%, p < .00001) and NC groups (3.4% vs 1%, p < .00001), although the difference did not reach statistical significance in the AHB group (3.2% vs 1.9%, p = .059). As previously reported [19, 20] and shown in **Fig 1B**, Vδ2^+^ γδT-cells largely co-expressed Vγ9 TCR and were CD3-bright without CD4 expression (i.e. CD3^hi^CD4^-^) unlike Vδ1^+^ γδT-cells which were not CD3-bright. Conversely, CD3^hi^CD4^-^ T-cell subset was highly enriched for Vδ2^+^ γδT-cells but not Vδ1^+^ γδT-cells, conventional αβTCR^+^ T-cells or CD1d-reactive NKT-cells, whereas CD3^int^CD4^-^ T-cells were enriched for conventional αβTCR^+^ and CD8^+^ T-cells but not Vδ2^+^ γδT-cells (**Fig 1C**). Accordingly, Vδ2^+^ γδT-cell frequency correlated significantly with frequencies of Vγ9^+^ γδT-cell and CD3^hi^CD4^-^ T-cells, but not Vδ1^+^ γδT-cells (**Fig 1D**). Thus, Vδ2^+^ γδT-cells with Vγ9 TCR co-expression and CD3^hi^CD4^-^ phenotype were the predominant circulating γδT-cells regardless of HBV infection.

Initial comparison of γδT-cell frequencies showed significantly higher CD3^hi^CD4^-^ and Vδ2^+^ (but not Vδ1^+^) γδT-cell frequencies in CHB compared to NC or AHB group (**Fig 1E**). However, since Asian Americans were highly enriched in CHB compared to NC or AHB group in our study (84% vs 47% vs 0%, p<0.001, **Table 1A**), we compared γδT-cell frequencies between Asian and non-Asian American subgroups in CHB and NC groups. As shown in **Fig 1F**, significant differences were detected between the 4 subgroups in circulating frequencies of Vδ2^+^ and CD3^hi^CD4^-^ γδT-cells but not Vδ1^+^ γδT-cells. In fact, Vδ2^+^ and CD3^hi^CD4^-^ γδT-cell frequencies were greater by 2–3 fold among Asian Americans compared to non-Asian Americans in both CHB group (%Vδ2^+^ γδT-cells: 8.6% vs 2.3%, p = .036; %CD3^hi^CD4^-^ γδT-cells: 3.5% vs 1.5%, p = .002) and NC group (%Vδ2^+^ γδT-cells: 6.7% vs 3%, p = .028; %CD3^hi^CD4^-^ γδT-cells: 2.5% vs 1.5%, p = .04).

As for another host factor, age showed significant inverse associations with Vδ2^+^ and CD3^hi^CD4^-^ (but not Vδ1^+^) γδT-cell frequencies (**Fig 1G**) consistent with lower γδT-cell frequencies reported in older persons [59, 60]. There were no differences between males or females (median %CD3^hi^CD4^-^/CD3: males 2.4% vs females 2.7%, p = .80). Finally, there were no significant correlations between γδT-cell frequencies and serum HBV DNA or alanine aminotransferase (ALT) levels in CHB subjects (**Fig 1H**). Thus, circulating γδT-cell frequencies were not altered by HBV infection in our study, although Vδ2^+^ γδT-cell frequency was significantly impacted by host factors such as race/ethnicity and age.

### Circulating γδT-cells display an innate phenotype with the expression of both T and NK regulatory markers that are distinctly altered in AHB and CHB

**Fig 2:**
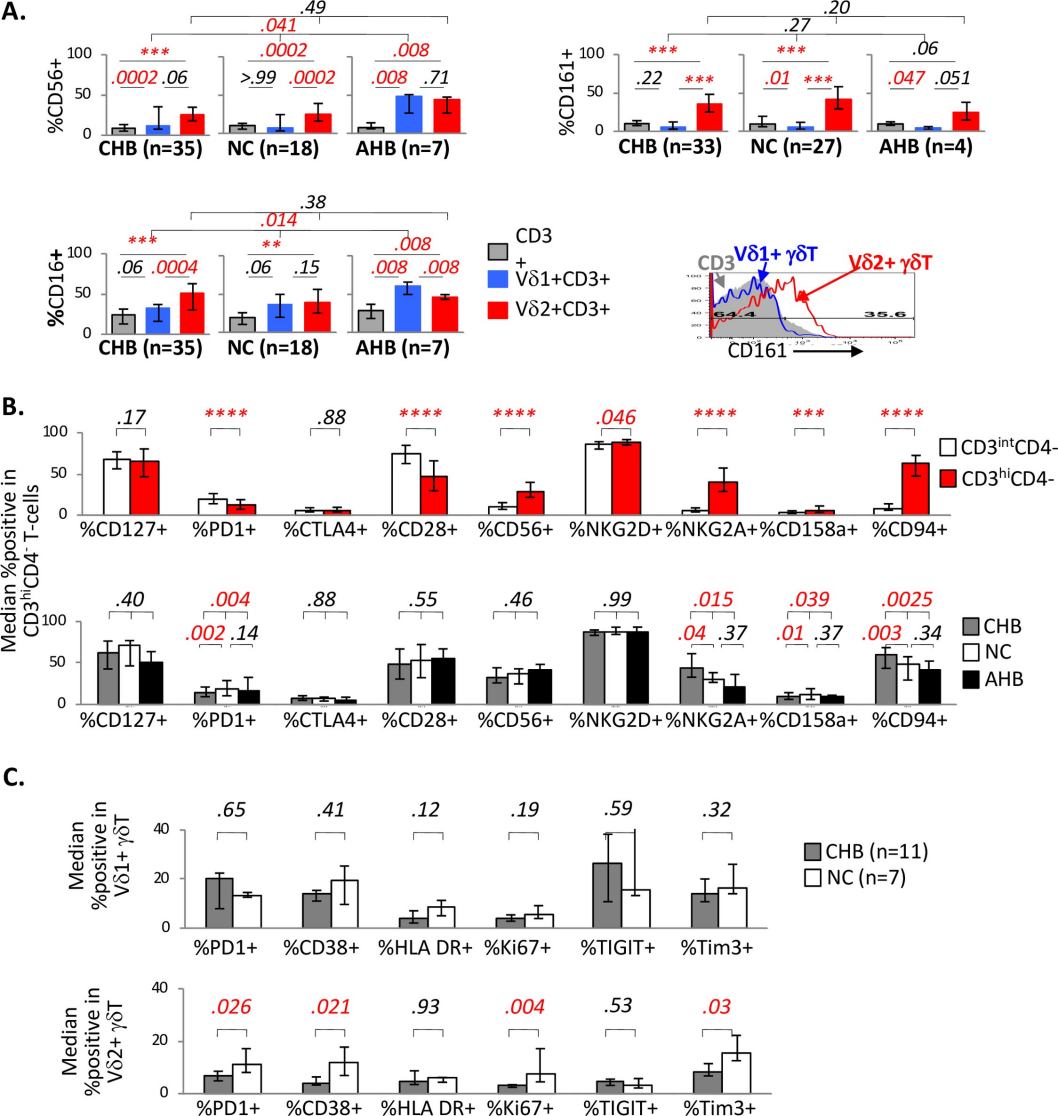
Circulating γδT-cells display an innate phenotype with the expression of both T and NK regulatory markers that are distinctly altered in AHB and CHB. **A. Innate phenotype of Vδ1**^**+**^ **a*v*δ/or Vδ2**^**+**^ **γδT-cells compared to total CD3**^**+**^ **T-cells.** Bar graphs show median %CD56^**+**^, %CD16^**+**^ and %CD161^**+**^ in total CD3^**+**^ T-cells (gray bars) relative to V**δ**1^**+**^ **γδ**T-cells (blue bars) or V**δ**2^**+**^ **γδ**T-cells (red bars) in CHB, NC and AHB groups, with error bars indicating 25% and 75% interquartile ranges. CD56 and CD16 expression levels were examined in 35 CHB, 18 NC and 7 AHB subjects, whereas CD161 expression was examined in 33 CHB, 27 NC and 4 AHB subjects. Expression levels between the cell subsets within individual subject were compared by matched pair signed-rank test. Comparisons between CHB, NC and AHB groups were made with Kruskal Wallis (k = 3). Histogram on the right bottom show overlay of CD3 (gray shade), V**δ**1^**+**^ **γδ**T-cells (blue line) or V**δ**2^**+**^ **γδ**T-cells (red line). **B. Increased expression of NK but not T-cell markers in CD3**^**hi**^**CD4**^**-**^ **T- compared to CD3**^**int**^**CD4**^**-**^ **T-cells.** (Top panel) Bar graphs show median % of cells expressing T-cell markers (CD127, PD-1, CTLA-4 and CD28) and NK markers (CD56, NKG2D, NKG2A, CD158a, CD94) in CD3^hi^CD4^-^ T-cells (red bars) and CD3^int^CD4^-^ T-cells (white bars) in CHB subjects, with p-values calculated by matched pair signed-rank test. (Bottom panel) Bar graphs compare median % of cells expressing T/NK markers in CD3^hi^CD4^-^ T-cells from CHB (gray bar), NC (white bar) and AHB (black bar) subjects. T-cell markers were measured in 189 CHB, 24 NC and 12 AHB subjects. NK markers were measured in 36 CHB, 17 NC and 7 AHB subjects. Error bars indicate 25% and 75% interquartile ranges. CHB, NC and AHB groups were compared by non-parametric Kruskal Wallis test (k = 3) with further comparisons between 2 groups by Mann Whitney U if the initial Kruskal Wallis test yielded p-values < 0.05. **C. Reduced expression of T-cell activation and exhaustion markers in Vδ2**^+^ **γδT cells from CHB compared to NC subjects**: Bar graphs compare 11 CHB (gray bars) and 7 NC subjects (white bars) for %Vδ1^+^ γδT cells (top) and %Vδ2^+^ γδT cells (bottom) that express various T-cell activation or exhaustion markers by CyTOF. Significant p-values < 0.05 are highlighted in red font. ****p < .0001; ***p < 0.0001; **p < .001.

Consistent with innate characteristics reported for γδT-cells [15], γδT-cells were more enriched in expression of NK markers compared to total CD3^+^ T-cells. For example, Vδ2^+^ γδT-cells expressed more CD56 and CD16 compared to total CD3^+^ T-cells, without significant differences between CHB, NC and AHB groups (**Fig 2A,** left panel). Vδ1^+^ γδT-cells also expressed more CD56 and CD16 than total CD3^+^ T-cells in AHB group compared to NC and CHB groups. As shown in **Fig 2A** (right panel), CD161, a C-type lectin expressed in NK cells and liver-homing T-cells with Th17/Th1 phenotype [61–64], was most enriched in Vδ2^+^ γδT-cells compared to other subsets, but without significant differences between CHB, NC and AHB groups.

Innate phenotype of Vδ2^+^ γδT-cells was further confirmed in CD3^hi^CD4 T-cells with significantly greater expression of NK-associated markers (e.g. CD56, NKG2A, CD94) but lower expression of T-cell associated markers (e.g. PD1, CD28), when compared to CD3^int^CD4^-^ T-cells (**Fig 2B,** top panel). Furthermore, compared to NC subjects, CHB subjects showed significantly lower expression of PD-1 and CD158a (inhibitory killer immunoglobulin-like receptor 2DL1 or KIR2DL1) but greater expression of inhibitory NKG2A and CD94 in CD3^hi^CD4^-^ T-cells, (**Fig 2B,** bottom panel). Further comparison of CHB and NC subjects with available lymphocytes confirmed reduced expression of PD1 as well as other activation and/or co-inhibitory markers including CD38, Ki67 and Tim3 in Vδ2^+^ γδT-cells from CHB compared to NC subjects, without such differences for Vδ1^+^ γδT-cells (**Fig 2C**). Collectively, these findings highlight distinct innate-like phenotype and activation of γδT-cells, with novel features of Vδ1^+^ γδT-cells in AHB (increased CD56 and CD16) and Vδ2^+^ γδT-cells in CHB (increased NKG2A/CD94 and reduced PD1, CD38, Ki67, Tim3 and CD158a,) compared to NC subjects.

### Tbet/Eomes expression is enriched in circulating Vδ1^+^ and Vδ2^+^ γδT-cells compared to total CD3^+^ T-cells, with differential hierarchy and phenotypes in CHB and AHB subjects

**Fig 3:**
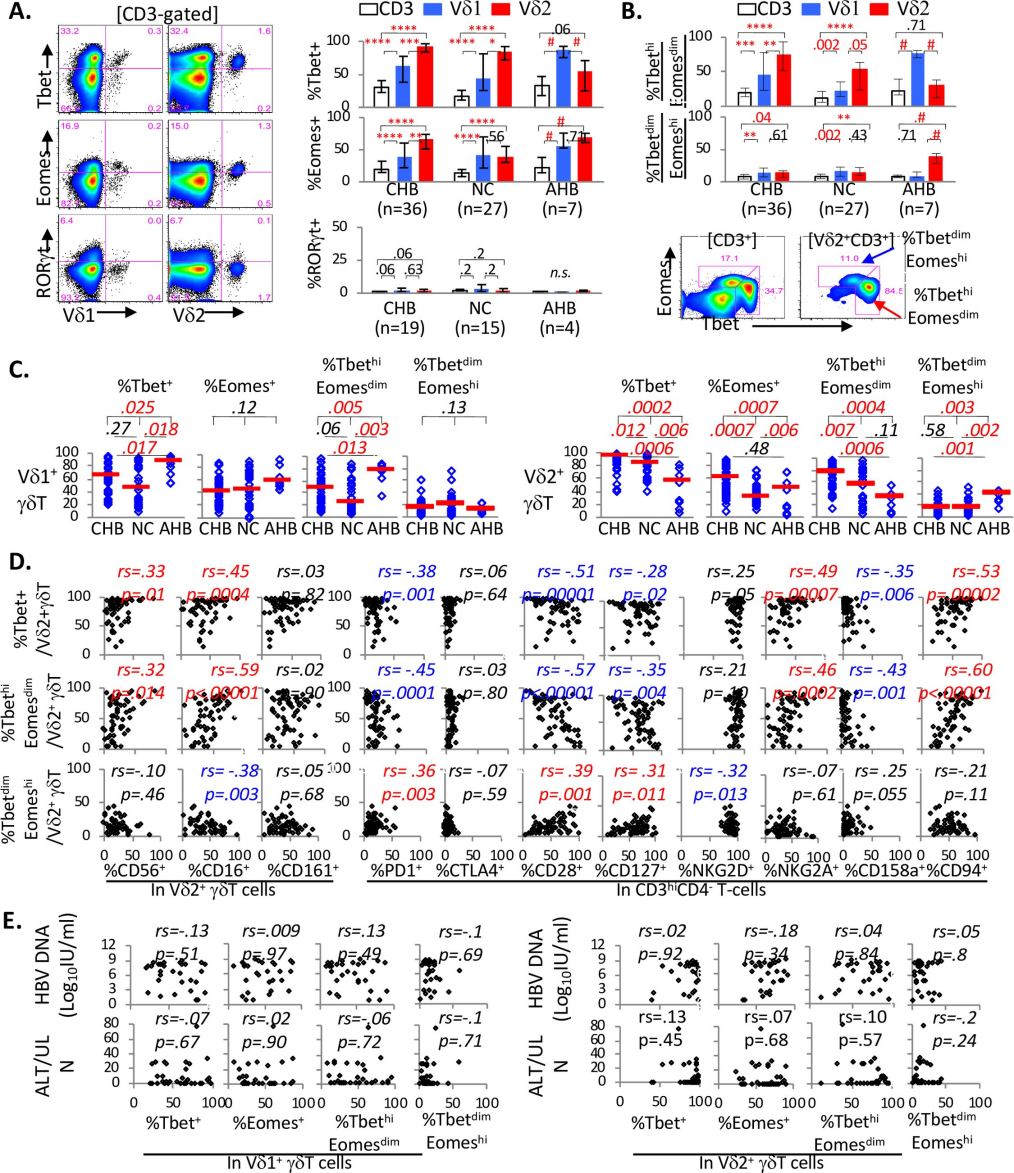
Tbet/Eomes expression is enriched in circulating Vδ1^+^ and Vδ2^+^ γδT-cells compared to total CD3+ T-cells with differential hierarchy and phenotypes in CHB and AHB subjects. **A. (Left panel) Representative FACS plots** showing Tbet, Eomes and RORγt expression in Vδ1^+^ γδT-cells and Vδ2^+^ γδT-cells in CD3-gated cells. (**Right panel**) **Comparisons of Tbet, Eomes and RORγt expression** between total CD3^+^ T-cells (white bars), Vδ1^+^ γδT-cells (blue bars) and Vδ2^+^ γδT-cells (red bars). Tbet and Eomes expression in T-cell subsets was examined in 36 CHB, 27 NC and 7 AHB subjects, with further examination of RORγt in 19 CHB, 15 NC and 4 AHB subjects based on PBMC availability. Bar graphs show median values within each group, with error bars indicating 25% and 75% interquartile ranges and p-values calculated by matched pair signed-rank test comparing T-cell subsets within each subject. In CHB group, the highest Tbet and Eomes expression was detected in Vδ2^+^ γδT-cells followed by Vδ1^+^ γδT-cells and total CD3^+^ T-cells. In NC group, similar hierarchy was detected for Tbet expression whereas Eomes expression was elevated in both Vδ1^+^ and Vδ2^+^ γδT-cells compared to total CD3^+^ T-cells. In AHB, Tbet was most elevated in Vδ1^+^ γδT-cells whereas Eomes was similarly elevated in both Vδ1^+^ and Vδ2^+^ γδT-cells compared to total CD3^+^ T-cells. RORγt was detected in very few cells in all groups, although statistical comparison was not possible for AHB subjects due to insufficient sample size (n = 4). ****p < 0.00001; ***p < .0001; **p < 0.001; *p < .01; #p = 0.008; n.s. sample size not sufficient for statistics for statistical comparison. **B. Comparison of Tbet**^**hi**^ **Eomes**^**dim**^ **and Tbet**^**dim**^ **Eomes**^**hi**^ **phenotype** between total CD3^+^ T-cells (white bars), Vδ1^+^ γδT-cells (blue bars) and Vδ2^+^ γδT-cells (red bars), with representative FACS density plot and gating strategy shown at the bottom for CD3^+^ T-cells (left) and Vδ2^+^ γδT-cells (right). ****p < 0.00001; ***p < .0001; **p < 0.001; *p < .01; #p = 0.008. **C. Comparison of Tbet/Eomes expression in circulating Vδ1**^**+**^ **and Vδ2**^**+**^ **γδT-cells from 36 CHB, 24 NC and 7 AHB subjects.** P-values between 3 groups were determined by Kruskal Wallis test (k = 3), followed by further two-way comparisons by Mann Whitney U for initial p-values below 0.05. **D. Correlations between Tbet/Eomes expression in Vδ2**^**+**^ **γδT-cells and their expression of NK and T-cell markers**. Percentages of Tbet^+^ or Tbet^hi^Eomes^dim^ Vδ2^+^ γδT-cells correlated positively with the expression of several NK markers (CD56, CD16, NNKG2A and CD94) and negatively with the expression of several T-cell markers (PD1, CD28, CD127) as well as KIR CD158a. Correlation coefficient and p-values were determined by non-parametric Spearman rank order correlation test. For convenience, red font was used to indicate significantly positive correlations with p-values <0.05 whereas blue font was used to indicate significantly negative correlations with p-values <0.05. **E. Lack of correlations between Tbet/Eomes expression in γδT-cells and serum HBV DNA or ALT activity.** Percentages of Tbet^+^, Eomes^+^, Tbet^hi^Eomes^dim^ and Tbet^dim^Eomes^hi^ Vδ1^+^ γδT-cells (left panel) or Vδ2^+^ γδT-cells (right panel) showed no significant correlations with serum HBV DNA or ALT activity. ULN (upper limit of normal) for ALT: 20 IU/L for females, 30 IU/L for males. Correlation coefficient and p-values were determined by non-parametric Spearman rank order correlation test.

As Vδ2^+^ γδT-cells were enriched in the expression of CD161, a marker associated with Th17/Th1 phenotype [61–64] }, we examined the expression of Th1 transcription factors Tbet and Eomes as well as Th17 transcription factor RORγt in Vδ2^+^ γδT-cells relative to those in Vδ1^+^ γδT-cells and CD3^+^ T-cells in CHB, NC and AHB subjects. In general, as shown in **Fig 3A**, Vδ1^+^ and Vδ2^+^ γδT-cells were more enriched in Tbet and/or Eomes expression than CD3^+^ T-cells, whereas RORγt was expressed in very few T-cells. As for reciprocal Tbet and Eomes expression associated with T-cell differentiation and/or exhaustion [65–68], both Tbet^hi^Eomes^dim^ and Tbet^dim^Eomes^hi^ populations were more prominent in γδT-cells than total CD3^+^ T-cells (**Fig 3B**). Both CHB and NC groups showed a hierarchy between T-cell subsets with the highest Tbet and/or Eomes expression as well as Tbet^hi^Eomes^dim^ phenotype in Vδ2^+^ γδT-cells followed by Vδ1^+^ γδT-cells and CD3^+^ T-cells. In AHB group, Vδ1^+^ γδT-cells were most enriched for Tbet^+^ and Tbet^hi^Eomes^dim^ cells, whereas Vδ2^+^ γδT-cells were most enriched in Tbet^dim^Eomes^hi^ cells associated with T-cell exhaustion [65]. Thus, Tbet/Eomes expression patterns were distinctly altered in circulating Vδ1^+^ and Vδ2^+^ γδT-cells from AHB and CHB subjects compared to uninfected controls.

As shown in **Fig 3C**, AHB subjects showed the greatest %Tbet^+^ and %Tbet^hi^Eomes^dim^ cells in Vδ1^+^ γδT-cells and least %Tbet^+^ with most Tbet^dim^Eomes^hi^ cells in Vδ2^+^ γδT-cells, compared to NC and CHB subjects. By contrast, CHB subjects showed the highest %Tbet^+^ and %Eomes^+^ as well as %Tbet^hi^Eomes^lo^ cells in Vδ2^+^ γδT-cells compared to NC and AHB subjects, without such differences for Vδ1^+^ γδT-cells. Our findings in Vδ2^+^ γδT-cells were not likely to reflect host race/ethnicity or age, since Tbet/Eomes expression patterns in Vδ2^+^ γδT-cells (e.g. CHB > NC for %Eomes^+^; AHB < NC and CHB for %Tbet^+^ and %Tbet^dim^Eomes^hi^) persisted among Asian and Non-Asian Americans despite reduced statistical significance with smaller sample sizes, and did not correlate with age (**S2A and S2B Fig**).

Notably, Tbet/Eomes expression in Vδ1^+^ and Vδ2^+^ γδT-cells correlated with their expression of NK markers CD56 and CD16, while CD161 expression correlated with Tbet/Eomes expression in Vδ1^+^ γδT-cells but not Vδ2^+^ γδT-cells (**Fig 3D, S3A Fig**). Furthermore, percentages of Tbet^+^ and Tbet^hi^Eomes^lo^ cells in Vδ2^+^ γδT-cells correlated significantly with the expression of various T and NK markers in CD3^hi^CD4^-^ and Vδ2^+^ γδT-cells, generally correlating inversely with T-cell markers (e.g. PD1, CD28, CD127) and directly with NK markers (NKG2A, CD94, CD56, CD16) with the exception of CD158a. Percentages of Tbet^dim^Eomes^hi^ Vδ2^+^ γδT-cells showed an opposite trend (directly with T-cell markers and inversely with NK markers). However, serum levels of HBV DNA or ALT in CHB subjects did not correlate with Tbet/Eomes expression in Vδ1^+^ or Vδ2^+^ γδT-cells (**Fig 3E**) or their expression of NK/T-cell markers (**S3B Fig**). Thus, circulating γδT-cells showed distinct patterns of Tbet/Eomes expression in CHB and AHB that further associated with their expression of various NK/T-cell markers, but not with clinical or virological measures in CHB.

### Circulating CD3^hi^CD4^-^ Vδ2^+^ γδT-cells show greater effector capacity compared to Vδ1^+^ γδT-cells and/or total CD3^+^ T-cells in-vitro

**Fig 4:**
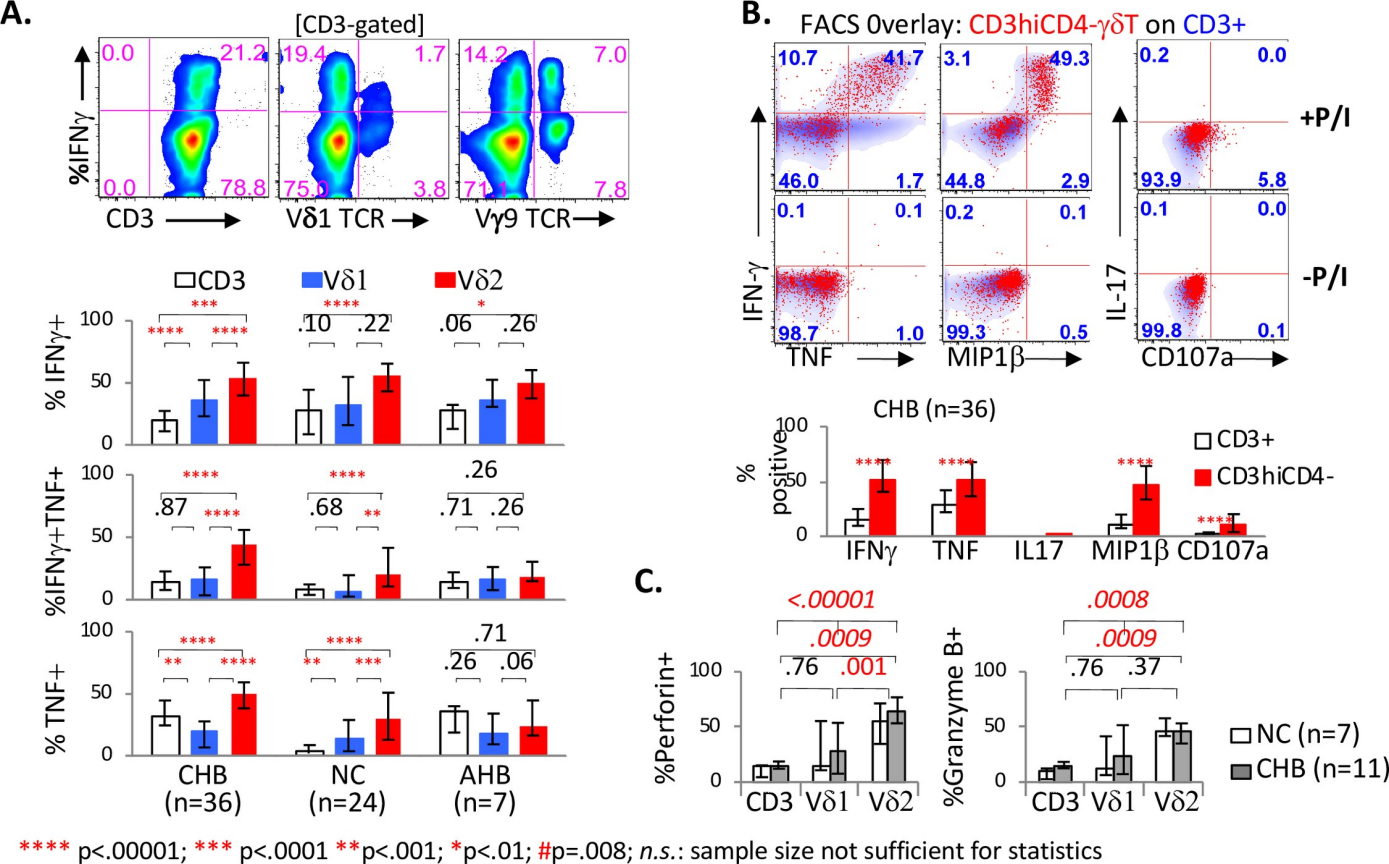
Circulating CD3^hi^CD4^-^ Vδ2^+^ γδT-cells show greater effector capacity compared to Vδ1^+^ γδT-cells and/or total CD3^+^ T-cells. **A. Hierarchy in IFNγ/TNF expression between total CD3**^**+**^ **T-cells, Vδ1**^**+**^ **γδT-cells and Vδ2**^**+**^ **γδT-cells from CHB, NC and AHB groups**. Upper panel shows representative FACS density plot appearance of CD3^+^ T-cells, Vδ1^+^ γδT-cells and Vδ2^+^ γδT-cells, with anti-Vγ9 TCR used to detect Vδ2^+^ (Vγ9^+^) γδT-cells. The 3 bar graphs below show median %IFNγ^+^, %IFNγ^+^TNF^+^ and %TNF^+^ cells in total CD3^+^ T-cells (white bars), Vδ1^+^ γδT-cells (blue bars) and Vδ2^+^ γδT-cells (red bars), with error bars indicating 25% and 75% interquartile ranges. P-values were calculated by non-parametric Mann Whitney U and shown above brackets to indicate the T-cell subsets being compared. Asterisks indicate significant p-values as follows: ****p < .00001; ***p < .0001; **p < .001; *p < .01. **B. CD3**^**hi**^**CD4**^**-**^ **T-cells show greater Th1 effector function compared to total CD3 T-cells**. Upper panel shows representative stainings for IFNγ, TNF, IL17+, MIP1β, and CD107a in FACS overlay of CD3^hi^CD4^-^ T-cells (red dots) onto CD3^+^ T-cells (blue shaded density) with and without 5 hours of PMA/Ionomycin (P/I) stimulation. Bar graphs on the lower panel show median %IFNγ+, %TNF+, %IL17+, %MIP1β+, %CD107a+ cells upon PMA/Ionomycin stimulation in gated CD3^hi^CD4^-^ T-cells (red bars) and total CD3 T-cells (white bars), with error bars indicating 25% and 75% interquartile ranges and p-values by Mann Whitney U test. **C. Vδ2**^**+**^ **γδT-cells from CHB subjects are enriched in effector molecules perforin and/or granzyme B compared to Vδ1**^**+**^ **γδT-cells or total CD3**^**+**^ **T-cells.** Bar graphs show median %Perforin^+^ and %Granzyme B^+^ in CD3^+^ T-cells, Vδ1^+^ γδT-cells and Vδ2^+^ γδT-cells examined ex vivo by CyTOF mass cytometry, with p-values by matched pair signed-rank test and error bars indicating 25% and 75% interquartile ranges.

We next compared the cytokine phenotype and effector capacity of Vδ1^+^ and Vδ2^+^ γδT-cells relative to total CD3^+^ T-cells by well-established intracellular cytokine staining protocol with brief 5 hours of PMA/ionomycin stimulation in-vitro [55–57]. As shown in **Fig 4A,** Vδ2^+^ γδT-cells showed the highest IFNγ response to PMA/Ionomycin in CHB, NC and AHB groups, with a significant hierarchy (Vδ2^+^ γδT-cells > Vδ1^+^ γδT-cells > total CD3^+^) in CHB although not other groups. Vδ2^+^ γδT-cells also showed the highest IFNγ/TNF co-expression and TNF expression in CHB and NC but not AHB group. Greater effector capacity in Vδ2^+^ γδT-cells was confirmed in CD3^hi^CD4^-^ T-cells compared to total CD3^+^ T-cells, by greater IFNγ, TNF and MIP1β expression as well as marginal CD107a mobilization (but without IL17 expression) (**Fig 4B**). Vδ2^+^ γδT-cells were also more enriched for cytolytic effector molecules perforin and granzyme B compared to Vδ1^+^ γδT-cells and/or CD3^+^T-cells (**Fig 4C**). Thus, circulating CD3^hi^CD4^-^ Vδ2^+^ γδT-cells showed greater effector capacity compared to other T-cell subsets, based on IFNγ/TNF responses to PMA/Ionomycin as well as perforin and granzyme B expression.

### IFNγ/TNF responses to brief PMA/Ionomycin stimulation are greater and more multi-functional in CD3^hi^CD4^-^ Vδ2^+^ γδT-cells from CHB compared to NC or AHB subjects

**Fig 5:**
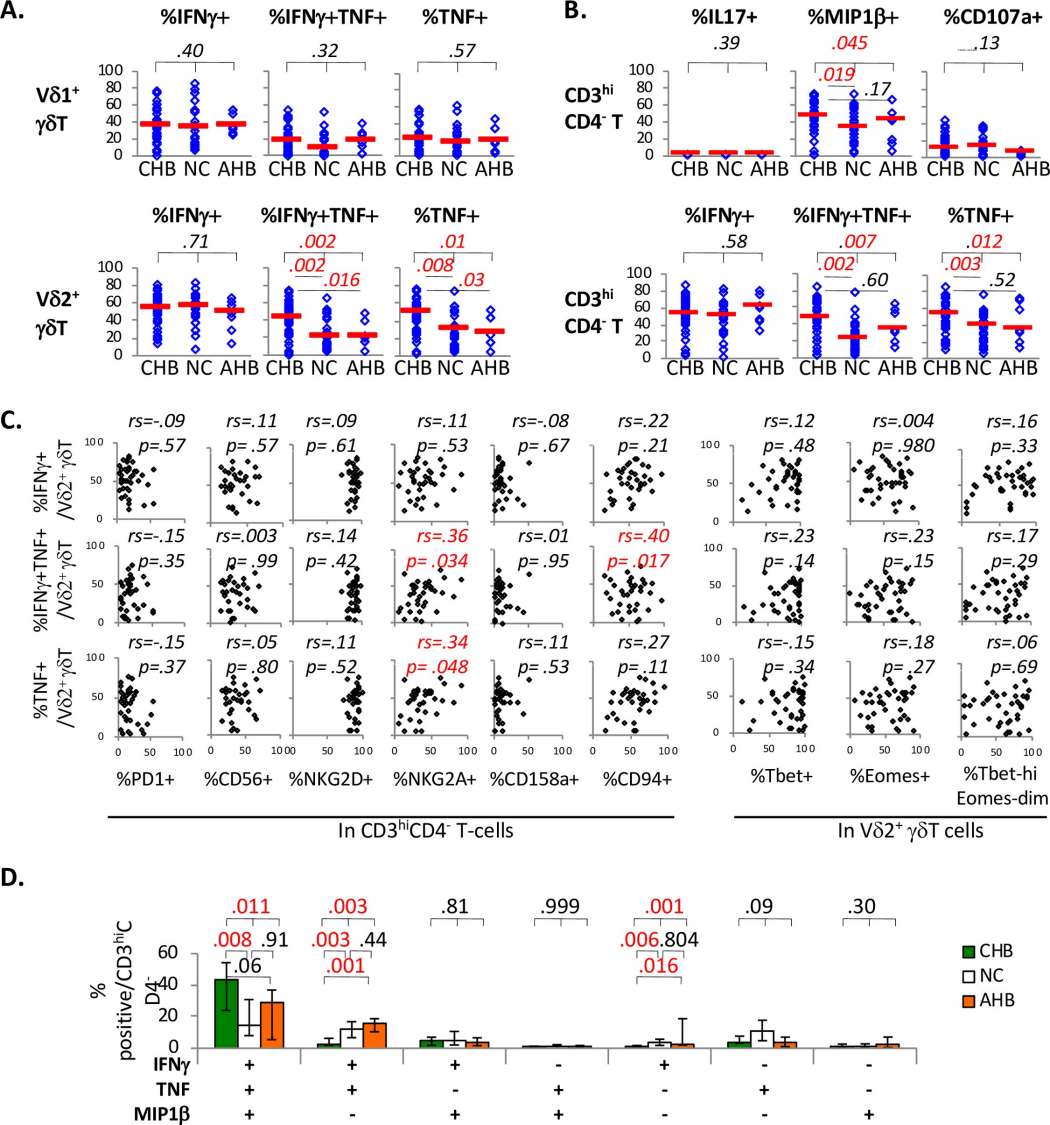
IFNγ/TNF responses to brief PMA/Ionomycin stimulation are greater and more multi-functional in CD3^hi^CD4^-^ Vδ2^+^ γδT-cells from CHB compared to NC or AHB subjects. **A/B. Comparison between CHB, NC and AHB groups of effector functions in circulating γδT-cell subsets.** CHB (n = 36), NC (n = 24) and AHB (n = 7) groups are compared for %IFNγ^+^, %IFNγ^+^/TNF^+^ and %TNF^+^ cells in Vδ1^+^ γδT-cells, Vδ2^+^ γδT-cells and CD3^hi^CD4^-^ T-cells following 5 hours of PMA/Ionomycin stimulation. P-values between 3 groups were determined by Kruskal Wallis test (k = 3), followed by further two-way comparisons by Mann Whitney U for initial p-value below 0.05. P-values below 0.05 were considered significant and highlighted in red font for convenience. **C. Correlations between early IFNγ/TNF responses in Vδ2**^**+**^ **γδT-cells to PMA/Ionomycin stimulation and their expression of T/NK markers.** Scatter plots show %IFNγ^+^, %IFNγ^+^/TNF^+^ and %TNF^+^ cells in Vδ2^+^ γδT-cells (following 5 hours of PMA/Ionomycin stimulation) on the y-axis, with x-axis showing percent expression of various T/NK markers as well as Tbet/Eomes. Correlation coefficient and p-values were determined by non-parametric Spearman rank order correlation test. For convenience, red font was used to indicate significantly positive correlations with p-values <0.05. **D. Comparison between CHB, NC and AHB groups for multi-functionality of CD3**^**hi**^**CD4**^**-**^ **T-cells following brief PMA/Ionomycin stimulation**. CD3^hi^CD4^-^ T-cells from CHB subjects (green bars) show greater co-expression of IFNγ, TNF and MIP1β following 5 hours of PMA/Ionomycin stimulation, compared to CD3^hi^CD4^-^ T-cells from NC (white bars) and AHB (orange bars) subjects. Error bars indicate 25% and 75% interquartile ranges. P-values between 3 groups were determined by Kruskal Wallis test (k = 3), followed by further two-way comparisons by Mann Whitney U for initial p-value below 0.05. P-values below 0.05 were considered significant and highlighted in red font for convenience. Gating strategy is shown in **S4 Fig**.

We further compared CHB, NC and AHB groups for IFNγ/TNF expression in γδT-cell subsets upon brief PMA/Ionomycin stimulation. As shown in **Fig 5A**, IFNγ and/or TNF response to PMA/Ionomycin did not differ between Vδ1^+^ γδT-cells from CHB, NC and AHB subjects. However, median %IFNγ^+^TNF^+^ double-positive and %TNF^+^ cells (but not %IFNγ^+^ cells) were significantly higher by almost 2 fold in Vδ2^+^ γδT-cells from CHB compared to NC and AHB subjects. The findings were similar between CHB and NC subjects for %IFNγ^+^TNF^+^ double-positive and %TNF^+^ cells as well as %MIP1β^+^ cells in CD3^hi^CD4^-^ T-cells, without differential CD107a mobilization or IL17 expression (**Fig 5B**). These differences in CD3^hi^CD4^-^ Vδ2^+^ γδT-cell function between CHB, NC and/or AHB groups were not due to race or age, as similar patterns persisted among Asian Americans for %IFNγ^+^TNF^+^ double-positive and %TNF^+^ cells (although not among Non-Asians with smaller sample sizes) and IFNγ/TNF responses did not correlate with age (**S2C and S2D Fig**).

As shown in **Fig 5C**, %TNF^+^ and/or %IFNγ^+^TNF^+^ cells in Vδ2^+^ γδT-cells showed significant positive correlations with their NKG2A and CD94 expression in CD3^hi^CD4^-^ T-cells, without significant correlations with PD1 or Tbet/Eomes expression. In further analysis of CD3^hi^CD4^-^ T-cells for multi-functionality based on IFNγ, TNF and/or MIP1β co-expression (**S4 Fig**), CHB subjects showed greater enrichment for IFNγ^+^TNF^+^MIP1β^+^ triple-positive CD3^hi^CD4^-^ T-cells following brief PMA/Ionomycin stimulation in-vitro, compared to NC and AHB subjects (42.3% vs 12.6% vs 23.9%, p = .025) (**Fig 5D**), with reciprocal reductions in IFNγ^+^TNF^+^MIP1β^-^ double positive (3.8% vs 9.3% vs 12.4%, p = .001) or IFNγ^+^TNF^-^MIP1β^-^ single positive cells (1.4% vs 3.7% vs 4.5%, p = .0002). Thus, functional responses to brief PMA/Ionomycin stimulation was preserved in circulating γδT-cells in HBV-infected patients, and even greater in Vδ2^+^ γδT-cells from CHB compared to NC and/or AHB subjects in association with NKG2A/CD94 expression.

### IFNγ/TNF responses to pAg are preserved in Vδ2^+^ γδT cells from CHB (but not AHB) subjects and are associated with their expression of Tbet/Eomes and NK markers but not PD1

**Fig 6:**
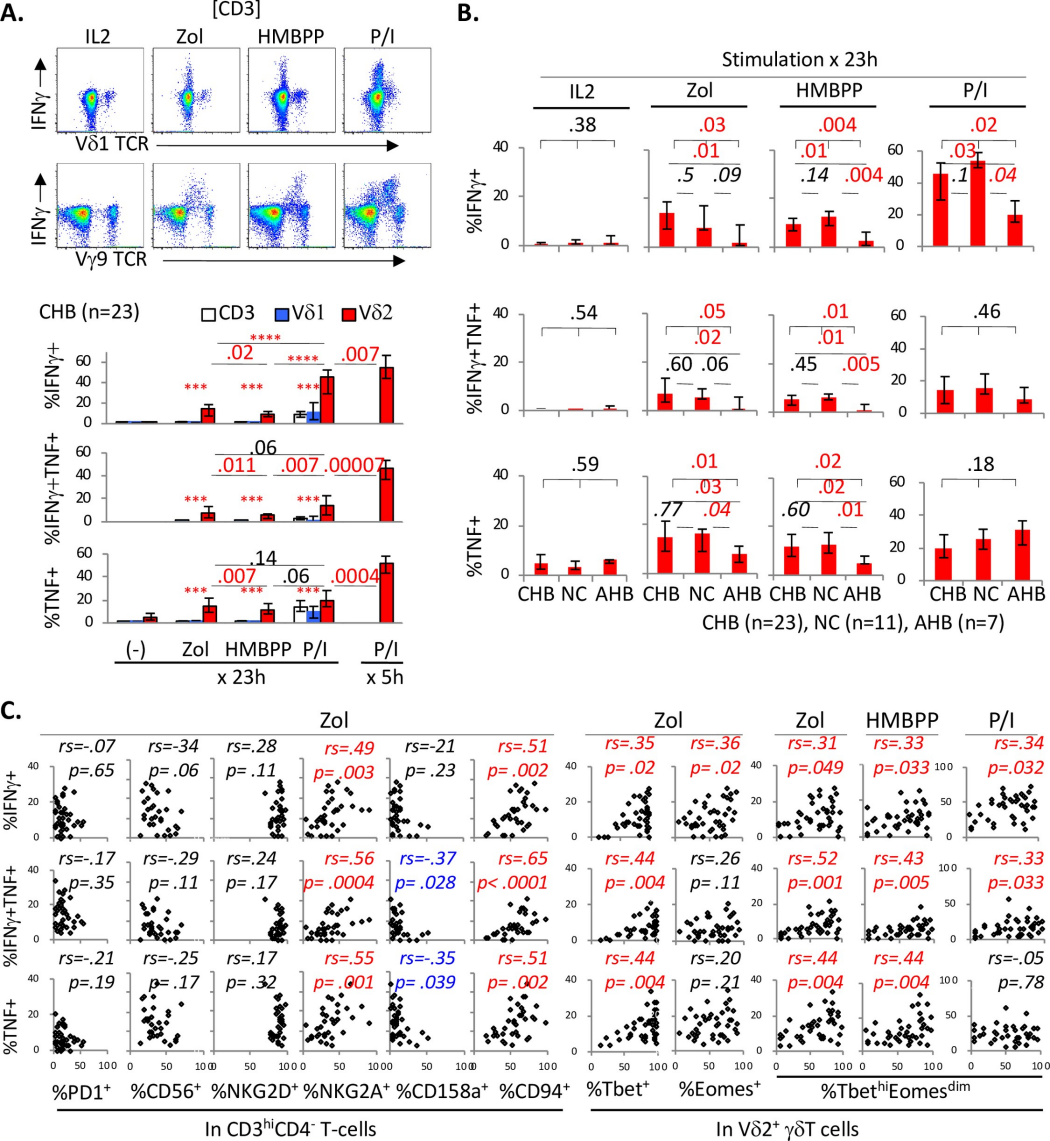
IFNγ/TNF responses to pAg are preserved in Vδ2+ γδT cells from CHB (but not AHB) subjects and are associated with their expression of Tbet/Eomes and NK markers but not PD1. **A. IFNγ/TNF responses to phosphoantigens in γδT-cells from CHB subjects.** Representative FACS plots show IFNγ expression in γδT-cells in CD3-gated cells. Anti-Vγ9 TCR was used to detect Vδ2^+^ (Vγ9^+^) γδT-cells. Bar graphs show %IFNγ^+^, %IFNγ^+^/TNF^+^ and %TNF^+^ cells in CD3^+^ T-cells (white bars), Vδ1^+^ γδT-cells (blue bars) and Vδ2^+^ γδT-cells (red bars) from 23 CHB subjects, with PBMC stimulated for 1 day (23 hours) in-vitro with phosphoantigens zoledronic acid (zol) or (E)-4-hydroxy-3-methyl-but-2-enyl pyrophosphate (HMBPP) in addition to PMA/Ionomycin. Red bars on the far right provide comparisons to early IFNγ/TNF responses in Vδ2^+^ γδT-cells stimulated for 5 hours in separate assays. Cytokine responses between CD3^+^ T-cells, Vδ1^+^ γδT-cells and Vδ2^+^ γδT-cells were compared by non-parametric Kruskal Wallis test (k = 3). Comparisons between 2 conditions were made with non-parametric Mann Whitney U. *** p < .0001; ****p < .00001. **B. Comparisons between CHB, NC and AHB subjects for IFNγ/TNF responses in Vδ2**^**+**^ **γδT-cells to 1 day stimulation with phosphoantigens and PMA/Ionomycin stimulation.** Bar graphs show %IFNγ^+^, %IFNγ^+^/TNF^+^ and %TNF^+^ cells in Vδ2^+^ γδT-cells following 1 day stimulation in-vitro with phosphoantigens zoledronic acid (zol) or (E)-4-hydroxy-3-methyl-but-2-enyl pyrophosphate (HMBPP) in addition to PMA/Ionomycin. Based on available cryopreserved PBMC, 23 CHB, 11 NC and 7 AHB subjects were included in this analysis. Error bars indicate 25% and 75% interquartile ranges. P-values between 3 groups were determined by Kruskal Wallis test (k = 3), followed by further two-way comparisons by Mann Whitney U for initial p-value below 0.05. P-values below 0.05 were considered significant and highlighted in red font for convenience. **C. Correlations between IFNγ/TNF responses in Vδ2**^**+**^ **γδT-cells to zoledronic acid and their expression of T/NK markers.** Scatter plots show %IFNγ^+^, %IFNγ^+^/TNF^+^ and %TNF^+^ cells in Vδ2^+^ γδT-cells (following 23 hours of stimulation) on the y-axis, with x-axis showing percent expression of various T/NK markers and Tbet/Eomes, combining results from 23 CHB, 11 NC and 7 AHB subjects. Correlation coefficient and p-values were determined by non-parametric Spearman rank order correlation test. For convenience, red font was used to indicate significantly positive correlations with p-values <0.05 whereas blue font was used to indicate significantly negative correlations with p-values <0.05.

While PMA and Ionomycin can provide robust pharmacological activation of multiple immune subsets [57] through protein kinase C and calcium signaling [69], phosphoantigens (pAg) such as zoledronate (Zol) or (E)-4-hydroxy-3-methylbut-2-enyl 4-diphosphate (HMBPP) specifically activate Vδ2^+^ γδT-cells as their natural ligands [17, 58, 70]. Based on control experiments in which IFNγ/TNF responses in Vδ2^+^ γδT-cells were greater with longer pAg stimulation for 23 hours compared to 8 hours (with opposite findings for PMA/Ionomycin) (**S1 Fig**), we examined the “late” cytokine responses in γδT-cells following stimulation for 23 hours with Zol, HMBPP and PMA/Ionomycin in-vitro. As shown in **Fig 6A**, IFNγ/TNF expression following Zol or HMBPP stimulation was detected in Vδ2^+^ γδT-cells (detected via Vγ9 TCR) but not Vδ1^+^ γδT cells or conventional CD3^+^ T-cells, with some downregulation of Vγ9 TCR upon activation in representative FACS plots on the top panel. Little to no IL-17 expression was detected in response to pAg or PMA/ionomycin, however (**S5 Fig**). Late IFNγ/TNF responses to pAg in Vδ2^+^ γδT-cells were also weaker than those induced by PMA/Ionomycin (both late and early as shown in far right in bottom bar graphs in **Fig 6A**).

As shown in **S6 Fig**, there were significant correlations between IFNγ/TNF responses in Vδ2^+^ γδT-cells to various stimulation conditions: 1) between late responses to Zol, HMBPP and PMA/Ionomycin; 2) between late responses to Zol and to HMBPP; 3) between late and early responses to PMA/Ionomycin. These correlations supported technical consistency of our assays as well as shared pathway whereby pAg and PMA/Ionomycin activate Vδ2^+^ γδT-cells. However, correlations were largely lost between early PMA/Ionomycin and late pAg responses, reflecting different kinetics and strengths whereby Vδ2^+^ γδT-cells are activated in-vitro by PMA/Ionomycin and pAg.

As shown in **Fig 6B**, AHB subjects showed significantly weaker late IFNγ/TNF responses in Vδ2^+^ γδT-cells to both Zol and HMBPP (as well as IFNγ response to PMA/Ionomycin) compared to NC or CHB subjects, but without significant differences between CHB and NC subjects detected in early responses to PMA/Ionomycin (**Fig 5A and 5B**). However, late IFNγ/TNF responses to pAg in Vδ2^+^ γδT-cells showed significant positive correlations with their NKG2A/CD94 expression (**Fig 6C**), similar to early IFNγ/TNF responses to PMA/Ionomycin (**Fig 5C**). Late IFNγ/TNF responses to pAg in Vδ2^+^ γδT-cells correlated positively with their Tbet/Eomes expression and inversely with CD158a expression, without significant associations with PD1 expression. Thus, late IFNγ/TNF responses to pAg in circulating Vδ2^+^ γδT-cells were weaker in AHB but preserved in CHB compared to uninfected controls, and correlated significantly with their expression of Tbet/Eomes and NK markers but not PD1.

### Serum ALT but not HBV DNA levels in CHB correlates inversely with IFNγ/TNF responses in Vδ1^+^ and Vδ2^+^ γδT-cells to brief PMA/Ionomycin stimulation, but not to pAg stimulation

**Fig 7:**
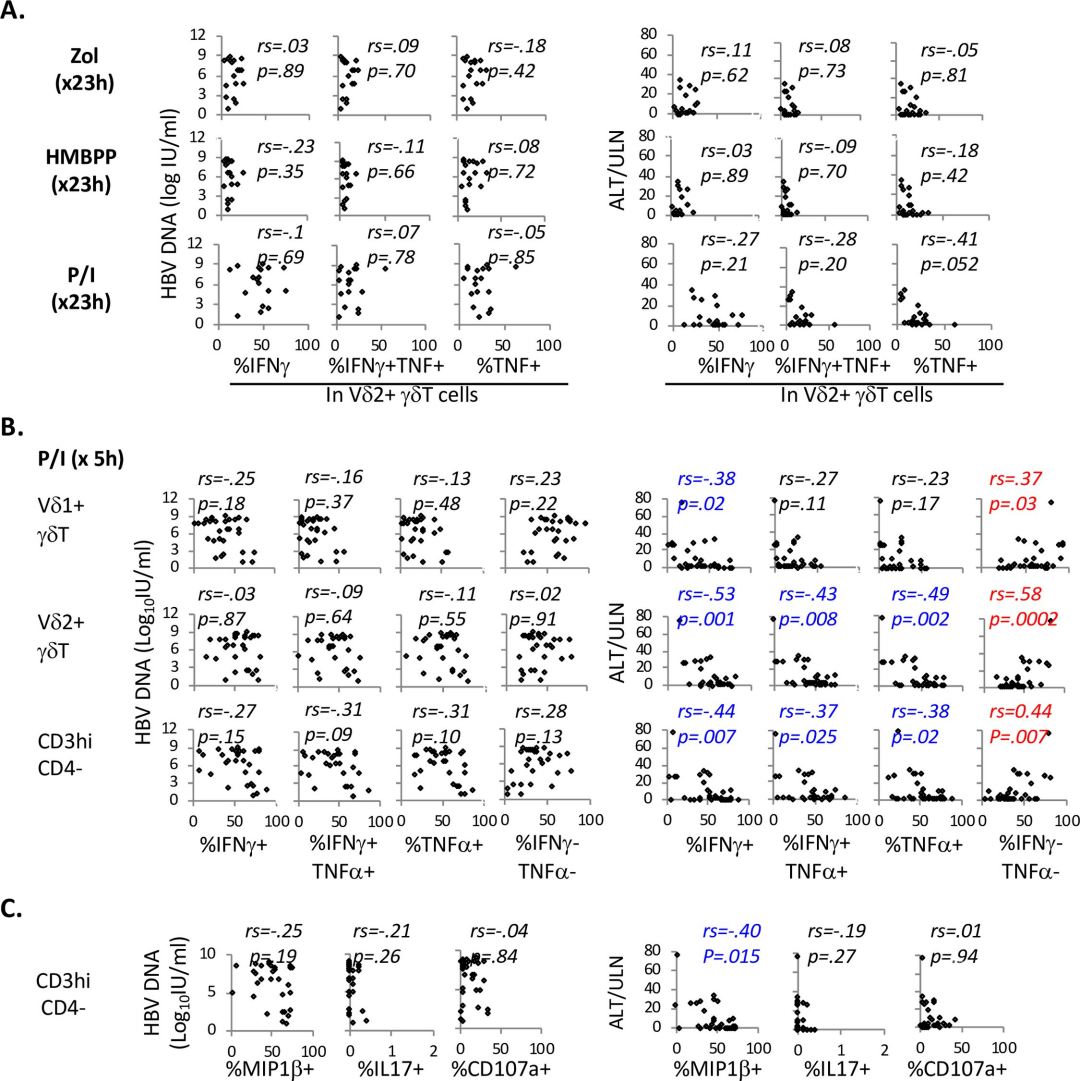
Serum ALT but not HBV DNA levels in CHB correlates inversely with IFNγ/TNF responses in Vδ1^+^ and Vδ2^+^ γδT-cells to brief PMA/Ionomycin stimulation, but not to pAg stimulation. **A. Serum HBV DNA and ALT levels in CHB do not correlate with IFNγ/TNF responses in Vδ2**^**+**^ **γδT-cells to 1 day stimulation with pAgs.** Scatter plots show %IFNγ^+^, %IFNγ^+^/TNF^+^ and %TNF^+^ cells in Vδ2^+^ γδT-cells on the x-axis, with y-axis showing concurrent levels of HBV DNA (log IU/ml) and ALT/ULN from the day of immune sample collection. Among 23 CHB subjects with available PBMC for pAg analysis, 23 had concurrent ALT values and 18 had concurrent HBV DNA levels for this analysis. Correlation coefficient and p-values were determined by non-parametric Spearman rank order correlation test. **B. Serum ALT (but not HBV DNA) levels in CHB correlate with early IFNγ/TNF responses in Vδ1**^**+**^ **γδT-cells, Vδ2**^**+**^ **γδT-cells and CD3**^**hi**^**CD4**^**-**^ **T-cells to PMA/Ionomycin stimulation.** Scatter plots show %IFNγ^+^, %IFNγ^+^/TNF^+^, %TNF^+^ and %IFNγ^-^/TNF^-^ cells in Vδ2^+^ γδT-cells on the x-axis, with y-axis showing concurrent levels of HBV DNA (log IU/ml) and ALT/ULN from the day of immune sample collection. Among 36 CHB subjects with available PBMC for early cytokine responses to PMA/Ionomycin, 36 had concurrent ALT values and 30 had concurrent HBV DNA levels. Correlation coefficient and p-values were determined by non-parametric Spearman rank order correlation test. For convenience, red font was used to indicate significant positive correlations with p-values <0.05 whereas blue font was used to indicate significant inverse correlations with p-values <0.05. **C. Serum ALT (but not HBV DNA) levels in CHB correlate with early MIP1**β **response in CD3**^**hi**^**CD4**^**-**^ **T-cells to PMA/Ionomycin stimulation.** Scatter plots show %MIP1β^+^, %IL17^+^, %CD107a^+^ CD3^hi^CD4^-^ T-cells on the x-axis, with y-axis showing concurrent levels of HBV DNA (log IU/ml) and ALT/ULN from the day of immune sample collection. Among 36 CHB subjects with available PBMC for early cytokine responses to PMA/Ionomycin, 36 had concurrent ALT values and 30 had concurrent HBV DNA levels. Correlation coefficient and p-values were determined by non-parametric Spearman rank order correlation test. For convenience, red font was used to indicate significantly positive correlations with p-values <0.05 whereas blue font was used to indicate significantly negative correlations with p-values <0.05.

We then examined if IFNγ/TNF responses in γδT-cells correlate with clinical or virological parameters in CHB. As shown in **Fig 7A**, late IFNγ/TNF responses to pAg or PMA/Ionomycin in Vδ2^+^ γδT-cells did not correlate significantly with serum HBV DNA or ALT. By contrast, early IFNγ/TNF responses to PMA/Ionomycin in Vδ2^+^ as well as CD3^hi^CD4^-^ γδT-cells showed significant inverse correlations with serum ALT (but not HBV DNA) (**Fig 7B**). Similar inverse correlation was detected between serum ALT and early IFNγ but not TNF expression in Vδ1^+^ γδT-cells following PMA/Ionomycin stimulation. Conversely, serum ALT correlated positively with %IFNγ^-^TNF^-^ double-negative Vδ1^+^, Vδ2^+^ or CD3^hi^CD4^-^ γδT-cells following brief PMA/Ionomycin stimulation. As shown in **Fig 7C**, early MIP1β expression following PMA/Ionomycin stimulation (but not CD107a mobilization or IL17 expression) by CD3^hi^CD4^-^ γδT-cells also correlated inversely with serum ALT, but not HBV DNA. Effect of hepatic fibrosis on γδT-cell phenotype and function could not be assessed as liver biopsy results were available in only 8/36 CHB subjects with detailed γδT-cell analyses, with only one showing cirrhosis. Thus, serum ALT was inversely associated with early IFNγ/TNF and MIP1β responses to PMA/Ionomycin in Vδ2^+^ γδT-cells (also IFNγ response in Vδ1^+^ γδT-cells) in-vitro, but not with late IFNγ/TNF responses to pAg or PMA/Ionomycin.

### CHB with ALT flare is associated with weaker early IFNγ/TNF responses to brief PMA/Ionomycin stimulation in Vδ2^+^ γδT-cells, compared to CHB without ALT flare

**Fig 8:**
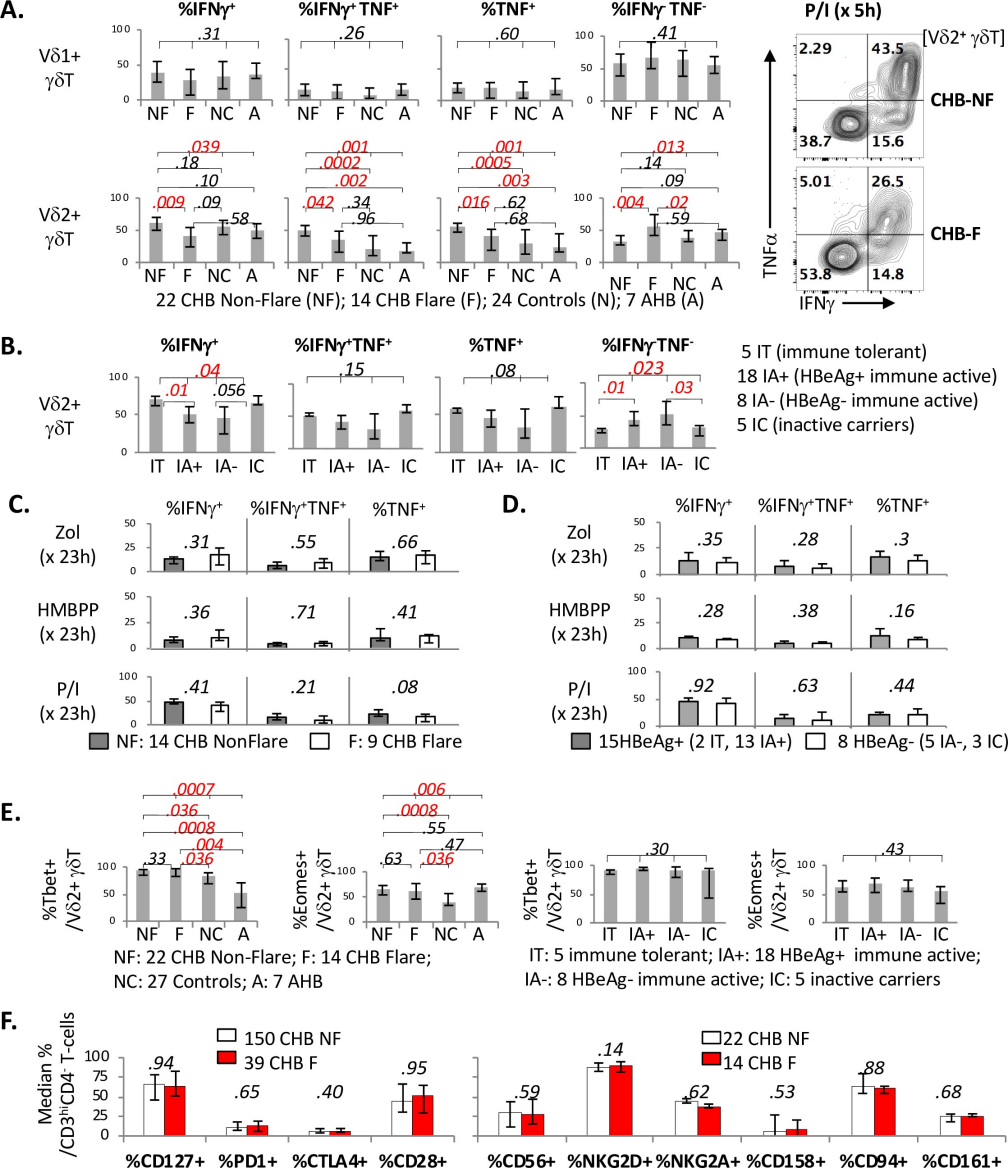
CHB with ALT flare is associated with weaker early IFNγ/TNF responses to brief PMA/Ionomycin stimulation in Vδ2^+^ γδT-cells, compared to CHB without ALT flare. **A. Early IFNγ/TNF responses to brief PMA/Ionomycin stimulation is greater in Vδ2**^**+**^ **γδT-cells (but not Vδ1**^**+**^ **γδT-cells) from CHB Non-Flare (NF) subjects compared to CHB Flare (F), NC or AHB subjects.** Bar graphs compare 22 CHB Non-Flare (NF), 14 CHB Flare (F), 24 uninfected control (NC) and 7 AHB (A) subjects for median percentage of cells with and without IFNγ/TNF expression in Vδ1^+^ γδT-cells (top panel) and Vδ2^+^ γδT-cells (bottom panel) upon 5 hours of PMA/Ionomycin stimulation in vitro. Right panel shows characteristic FACS contour plots for IFNγ and TNF expression in Vδ2^+^ γδT-cells upon PMA/Ionomycin stimulation from CHB-Non-Flare and CHB-Flare subjects. Error bars indicate 25% and 75% interquartile ranges. P-values between 4 subgroups were determined by Kruskal Wallis test (k = 4), followed by further two-way comparisons by Mann Whitney U for initial p-value below 0.05. P-values below 0.05 were considered significant and highlighted in red font for convenience. **B. Early IFNγ/TNF response to brief PMA/Ionomycin stimulation is greater in Vδ2**^**+**^ **γδT-cells from clinical CHB phenotype groups with lower ALT values.** Bar graphs compare 5 immune tolerant (IT), 18 HBeAg+ immune active (IA+), 8 HBeAg- immune active (IA-) and 5 inactive carrier (IC) subjects with CHB, showing median percentage of cells with and without IFNγ/TNF expression in Vδ2^+^ γδT-cells following 5 hours of PMA/Ionomycin stimulation in vitro. Error bars indicate 25% and 75% interquartile ranges. P-values between 4 subgroups were determined by Kruskal Wallis test (k = 4), followed by further two-way comparisons by Mann Whitney U for initial p-value below 0.05. P-values below 0.05 were considered significant and highlighted in red font for convenience. **C/D. IFNγ/TNF response to 1 day of pAg stimulation does not differ between CHB Non-Flare (NF) subjects compared to CHB Flare (F) or between HBeAg+ or HBeAg- CHB subjects.** Bar graphs compare 14 CHB Non-Flare (NF) and 9 CHB Flare (F) subjects, as well as 15 HBeAg+ CHB and 8 HBeAg- CHB subjects, with median percentage of cells with and without IFNγ/TNF expression in Vδ2^+^ γδT-cells following 1 day (23 hours) stimulation with phosphoantigens zoledronic acid (zol) or (E)-4-hydroxy-3-methyl-but-2-enyl pyrophosphate (HMBPP) in addition to PMA/Ionomycin (P/I). P-values between 2 subgroups were determined by Mann Whitney U with p-values below 0.05 considered significant. E. **Lack of differential Tbet and Eomes expression between Vδ2**^+^ **γδT-cells from CHB Non-Flare and CHB Flare subjects.** Bar graphs compare median %Tbet+ and %Eomes+ cells in Vδ2^+^ γδT-cells between patient groups, without significant differences between CHB Non-Flare and CHB Flare subjects. F. **Lack of differential T/NK marker expression in CD3**^**hi**^**CD4**^**-**^ **Vδ2**^+^ **γδT-cells from CHB Non-Flare and CHB Flare subjects.** Bar graphs compare median % of cells expressing T/NK markers in CD3^hi^CD4^-^ or Vδ2^+^ γδT-cells from CHB Non-Flare (CHB NF, white bar) and CHB Flare (CHB F, red bar) subjects. Expression of T-cell markers (CD127, PD1, CTLA4 and CD28) in CD3^hi^CD4^-^ T-cells were measured in 150 CHB NF and 39 CHB F subjects. Expression of NK markers (CD56, NKG2D, NKG2A, CD158a and CD94) in CD3^hi^CD4^-^ T-cells were measured in 22 CHB NF and 14 CHB F subjects. CD161 expression was measured in Vδ2^+^ γδT-cells from 22 CHB NF and 9 CHB F subjects. Error bars indicate 25% and 75% interquartile ranges. CHB, NC and AHB groups were compared by non-parametric Kruskal Wallis test (k = 3) with further comparisons between 2 groups by Mann Whitney U if the initial Kruskal Wallis test yielded p-values < 0.05.

ALT flares in CHB are clinically relevant events associated with necroinflammatory changes in the liver [71–73]. Given the inverse associations between serum ALT and early IFNγ/TNF responses in γδT-cells to PMA/Ionomycin stimulation in CHB, CHB subjects with and without a recent hepatitis flare (ALT/ULN ≥10 within a month of immune analyses) were further compared for IFNγ/TNF responses in γδT-cells. As shown in **Fig 8A**, early IFNγ/TNF responses in Vδ2^+^ (but not Vδ1^+^) γδT-cells to PMA/Ionomycin were significantly greater in CHB Non-Flare (NF) compared to CHB Flare (F), NC and/or AHB subjects. Conversely, IFNγ^-^TNF^-^ double-negative cells were more enriched in Vδ2^+^ γδT-cells from CHB F compared to CHB NF and NC subjects.

Further comparisons between clinically defined CHB phenotype subgroups showed significantly greater early IFNγ/TNF responses to PMA/Ionomycin in Vδ2^+^ γδT-cells from immune tolerant (IT) or inactive carrier (IC) groups with normal ALT, compared to HBeAg+ immune active (IA+) or HBeAg- immune active (IA-) groups with elevated ALT (**Fig 8B**). As for late IFNγ/TNF responses in Vδ2^+^ γδT-cells to pAg or PMA/Ionomycin stimulation, comparisons between CHB IT, IA or IC subgroups could not be made due to small sample sizes precluding meaningful statistical comparisons (e.g. only 2 IT and 3 IC), although no significant differences were detected between CHB subgroups with and without ALT flares (**Fig 8C**) or with and without circulating HBeAg (**Fig 8D**).

In further comparisons, Tbet/Eomes expression in Vδ2^+^ γδT-cells did not differ significantly between CHB Non-Flare and Flare or between clinical CHB phenotype subgroups (**Fig 8E**), although both CHB Non-Flare and Flare subgroups showed significantly greater Tbet/Eomes expression in Vδ2^+^ γδT-cells compared to NC and AHB groups. Otherwise, CHB Non-Flare and Flare subgroups did not differ significantly in the expression of immune regulatory markers such as PD-1, CTLA-4, CD28, CD127, CD56, CD94, CD158a, NKG2A, NKG2D and CD161 in CD3^hi^CD4^-^ T-cells (**Fig 8F**). Thus, compared to CHB Non-Flare subjects, CHB Flare subjects showed weaker early IFNγ/TNF responses in Vδ2^+^ γδT-cells to PMA/Ionomycin, but without differential expression of Tbet/Eomes or T/NK regulatory markers.

### Early IFNγ/TNF responses in Vδ2^+^ γδT-cells to brief PMA/Ionomycin stimulation improve with the resolution of AHB but not CHB Flare

**Fig 9:**
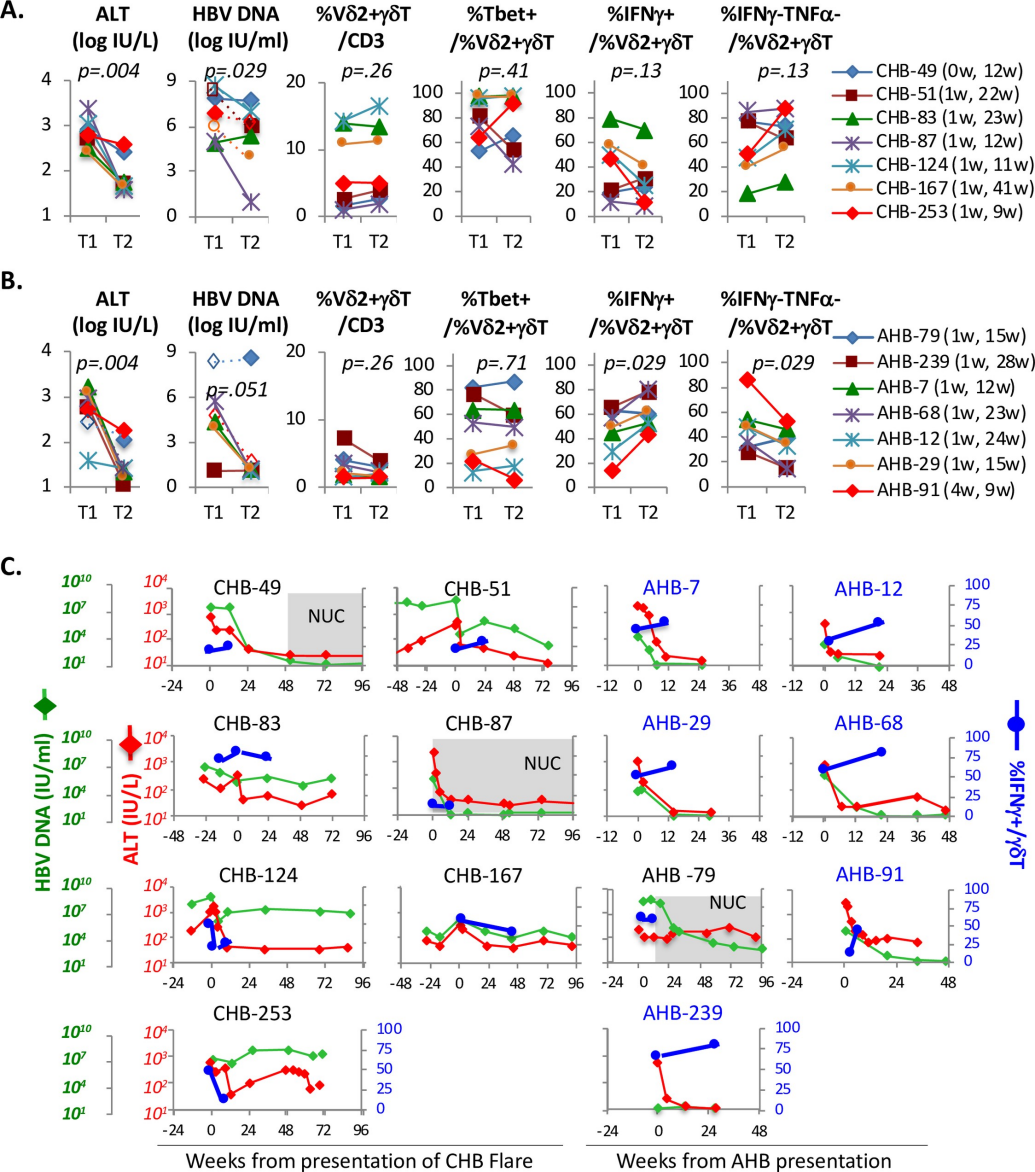
Early IFNγ/TNF responses in Vδ2^+^ γδT-cells to brief PMA/Ionomycin stimulation improve with the resolution of AHB but not CHB Flare. **A/B. Clinical, virological and immunological measures during and after CHB Flare or AHB.** Graphs compare serum ALT (log IU/L), HBV DNA (log IU/L), %Vδ2^+^ γδT-cells/CD3^+^ T-cells, %Tbet/Vδ2^+^ γδT-cells, %IFNγ^+^/Vδ2^+^ γδT-cells, %IFNγ^-^TNF^-^/Vδ2^+^ γδT-cells for 7 CHB-Flare subjects **(A)** and 7 AHB subjects **(B)** at the earliest time point (T1) within 1 weeks from initial clinical presentation for CHB flare or AHB and a later time point T2 with resolution of ALT flare or AHB. Subject ID and the timing of T1 and T2 blood draws for immune analyses (relative to clinical presentation) are shown on far right. Dotted lines with unfilled markers indicate that ALT or HBV DNA values were missing from those time points and substituted from the closest available time points. Cryopreserved PBMC from T1 and T2 time points for each subject were assayed concurrently for better comparability. As shown, ALT and HBV DNA levels declined by T2 in most subjects. No significant changes were detected for %Vδ2^+^ γδT-cells/CD3^+^ T-cells or %Tbet/Vδ2^+^ γδT-cells. AHB (but not CHB) subjects showed increased %IFNγ^+^/Vδ2^+^ γδT-cells and decreased %IFNγ^-^TNF^-^/Vδ2^+^ γδT-cells between T1 and T2. **C. Evolution of serum ALT and HBV DNA levels relative to IFNγ expression in Vδ2**^**+**^ **γδT-cells in CHB Flare and AHB subjects.** Serum HBV DNA (green diamond) and ALT (red diamond) levels are shown over time (in weeks from presentation of CHB flare or AHB), and juxtaposed to %IFNγ^+^/Vδ2^+^ γδT-cells (blue filled circle) for 7 CHB Flare and 7 AHB subjects. Notably, %IFNγ^+^/Vδ2^+^ γδT-cells increased between T1 and T2 in AHB but not CHB subjects.

To determine if clinical resolution of ALT flare in CHB is associated with changes in Vδ2^+^ γδT-cells, 7 CHB F subjects with available cryopreserved PBMC were examined at a second time point (T2) at least 2 months (9–41 weeks) after the initial evaluation (T1) for frequency, phenotype and early IFNγ responses to PMA/Ionomycin (**Fig 9A**). Seven AHB subjects with available PBMC at similar time frame were also examined for comparison (**Fig 9B**). As expected, ALT levels declined after the initial evaluation in all subjects, whereas HBV DNA levels declined in most but not all subjects. However, the overall circulating frequency and Tbet expression in Vδ2^+^ γδT-cells did not differ significantly between T1 and T2 in CHB Flare or AHB subjects. Furthermore, early IFNγ responses to PMA/Ionomycin in Vδ2^+^ γδT-cells did not change significantly between T1 and T2 for CHB Flare subjects (median %IFNγ^+^: T1 46.9% vs T2 25.9%, p = .13) and in fact declined in 5/7 subjects. By contrast, IFNγ expression in Vδ2^+^ γδT-cells increased in 6/7 AHB subjects (median %IFNγ+: T1 49.5% vs T2 60.2%, p = .029). Conversely, %IFNγ^-^TNFα^-^ double-negative Vδ2^+^ γδT-cells declined significantly in AHB but not CHB Flare subjects.

We further examined the dynamic evolution in serum ALT (red diamond) and HBV DNA (green diamond) relative to %IFNγ^+^/Vδ2^+^ γδT-cells (blue circle) in CHB Flare and AHB subjects (**Fig 9C**). As shown, resolution of ALT flare was not associated with increased %IFNγ^+^/Vδ2^+^ γδT-cells in most CHB Flare subjects, including CHB-87 who achieved prompt reduction in ALT and HBV DNA on antiviral therapy (gray shade). By contrast, %IFNγ^+^/Vδ2^+^ γδT-cells increased in most AHB subjects as acute ALT elevation and viremia resolved, with the exception of AHB-79 who initiated antiviral therapy with prolonged viremia above 4 log and ALT elevation beyond 24 weeks.

Collectively, we show that γδT-cells are preserved in circulating frequency but altered with distinct innate phenotype and effector function in acute and chronic HBV infection with relevance to clinical status.

## Discussion

While T-cells play a critical role in disease pathogenesis and viral clearance in acute HBV infection, their role in chronic HBV infection is less clear due to functional impairment with the induction of multiple immune regulatory pathways [4–12]. As γδT-cells are non-conventional T-cells that participate in lymphoid stress surveillance [29, 30] and microbial pathogenesis [17], we asked if γδT-cells contribute to HBV pathogenesis. To this end, we examined the frequency, phenotype and effector function of circulating γδT-cells in a North American cohort of HBV-infected and uninfected subjects. Our findings show that γδT-cells are preserved in circulating frequency regardless of HBV infection, with CD3^hi^CD4^-^ Vδ2^+^ γδT-cells as the predominant subset. We further show distinct phenotypic and functional characteristics of γδT-cells in acute and chronic HBV infection with potential pathogenetic relevance.

Contrary to altered γδT-cell frequencies reported in HIV, CMV and several other intracellular pathogens such as mycobacterium tuberculosis [17, 36–39], circulating γδT-cell frequencies did not differ significantly between HBV-infected and uninfected subjects in our study. Our findings differ from reports associating γδT-cell frequencies with clinical status of CHB [44–48]. For example, reduced Vδ2^+^ γδT-cell frequency was associated with severe HBV-associated liver disease in several studies of Chinese subjects with CHB [44–46]. Conversely, asymptomatic HBV carriers with low HBV DNA and ALT showed increased circulating Vδ1^+^ and Vδ2^+^ γδT-cell frequencies compared to controls in a study from Ireland with mixed African, Caucasian and Asian subjects [48]. Unlike previous studies, we examined North Americans well-preserved liver function without decompensation [10, 50]. Although most of our CHB subjects consisted of Asian Americans, our findings persisted when Asian and non-Asian Americans were examined separately. We also confirmed circulating γδT-cell frequencies by multiple approaches (e.g. staining for Vδ2 TCR and Vγ9 TCR as well as CD3^hi^CD4^-^ phenotype). Furthermore, our cohort included diverse clinical CHB phenotypes including immune tolerant, immune active and inactive carriers as well as those with ALT flares, supporting the generalizability of our findings.

There was a notable impact of host factors for Vδ2^+^ γδT-cell frequency. For example, Asian Americans displayed 2–3 fold higher circulating Vδ2^+^ γδT-cell frequency compared to Non-Asian Americans. These differences were not due clinical or virological status of CHB as they were also detected in uninfected controls. They were not associated with HIV or CMV co-infection, since HIV-infected or immunosuppressed subjects were excluded in our study and CMV infection generally impacts Vδ1^+^ (not Vδ2^+^) γδT-cells [40–42]. To our knowledge, differential γδT-cell frequency between Asian and Non-Asian Americans has not been reported, although greater Vδ2^+^ γδT-cell frequency was described in Caucasian compared to African Americans [74]. The inverse association between age and Vδ2^+^ (but not Vδ1^+^) γδT-cell frequency was consistent with previous reports [59, 60]. The underlying mechanisms that govern γδT-cell homeostasis and their clinical implications are not well-defined, although both environmental factors (e.g. diet, infection, microbes) as well as genetics are shown to impact human immune system [75–77]. In fact, Vδ2^+^Vδγ9^+^ γδT-cells showed greater heritability than other innate-line T-cells such as Vδ1^+^ γδT-cells and NKT-cells, although innate immune traits were influenced more by environment whereas adaptive immune traits were impacted more by genetic [78]. In any case, the marked differences in Vδ2^+^ γδT-cell frequency between Asians and Non-Asians suggest that race/ethnicity must be considered in clinical studies involving Vδ2^+^ γδT-cells, including immunotherapy [31–35].

Another key finding in our study is the distinct innate phenotype with increased Tbet/Eomes expression in circulating γδT-cells in AHB and CHB compared to uninfected control subjects. In general, Vδ1^+^ and Vδ2^+^ γδT-cells expressed more Tbet/Eomes and NK markers compared to total CD3^+^ T-cells (e.g. Vδ2^+^ > Vδ1^+^ > total CD3^+^ T-cells for %Tbet and %Tbet^hi^Eomes^dim^ in both CHB and NC). However, compared to NC and CHB subjects, AHB subjects displayed more effector-like Tbet^hi^Eomes^dim^ Vδ1^+^ γδT-cells but also more exhausted Tbet^dim^Eomes^hi^ Vδ2^+^ γδT-cells [65, 66] (**Fig 3C**). Furthermore, AHB subjects displayed more CD56 and CD16 expression in Vδ1^+^ (but not Vδ2^+^) γδT-cells compared to NC and CHB subjects (**Fig 2A**). On the other hand, CHB subjects showed significantly greater Tbet expression and Tbet^hi^Eomes^dim^ phenotype in CD3^hi^CD4^-^ Vδ2^+^ γδT-cells compared to NC subjects, without such differences for Vδ1^+^ γδT-cells (**Fig 3C**). Furthermore, compared to uninfected control subjects, CHB subjects expressed less PD1, Tim3, CD38, Ki67 and CD158a but more NKG2A and CD94 in CD3^hi^CD4^-^ Vδ2^+^ γδT-cells (**Fig 2B and 2C**). Thus, circulating γδT-cells in AHB and CHB showed altered differentiation based on Tbet/Eomes expression, as well as activation through innate pathways involving NK receptors but not T-cell regulatory pathways including PD1.

Tbet/Eomes expression patterns in γδT-cells correlated significantly with their expression of NK and T-cell activation or co-stimulatory markers, but not serum ALT or HBV DNA levels. For example, percentages of Tbet^+^, Eomes^+^ or Tbet^hi^Eomes^dim^ cells in Vδ1^+^ γδT-cells showed significant positive correlations with their CD56, CD16 and CD161 expression (**S3 Fig**). Percentages of Tbet^+^ or Tbet^hi^Eomes^dim^ in Vδ2^+^ γδT-cells also correlated positively with their expression of NK markers such as NKG2A, CD94, CD56 and CD16, but inversely with T-cell markers (e.g. PD-1, CD28 and CD127) as well as a killer immunoglobulin-like receptor CD158a (**Fig 3D**). Inverse associations between Tbet and PD1 as well as other co-inhibitory receptors have been reported in CD8 T-cells whereby Tbet mediates direct transcriptional repression of PD1 [79]. To our knowledge, similar associations between Tbet and PD-1 as well as other T/NK markers have not been reported in γδT-cells, but suggest a critical regulatory role for Tbet in γδT-cells. These broad and inter-related phenotypic alterations in circulating γδT-cells from AHB and CHB patients also suggest that γδT-cells participate in lymphocyte stress surveillance during HBV infection.

Several new findings emerged in our study regarding γδT-cell function in HBV infection, despite different kinetics and strengths whereby Vδ2^+^ γδT-cells were stimulated in-vitro by pAg (through the TCR) or non-specifically by PMA/Ionomycin (through protein kinase C and calcium signaling) [16, 26, 69, 80]. First, compared to NC subjects, CHB subjects showed well-preserved IFNγ/TNF responses in circulating Vδ1^+^ and Vδ2^+^ γδT-cells (even greater for early PMA/Ionomycin responses in Vδ2^+^ γδT-cells). This contrasted from functional impairment reported for conventional T-cells and B-cells in CHB [10, 11, 81, 82], but resemble recent findings in HCV-infected patients [64]. Second, AHB subjects displayed significantly weaker IFNγ/TNF responses in Vδ2^+^ γδT-cells compared to CHB subjects for early PMA/Ionomycin response, and compared to both CHB and NC subjects for late responses to pAg and PMA/Ionomycin. By contrast, Vδ1^+^ γδT-cells from AHB subjects showed preserved early IFNγ/TNF responses to PMA/ionomycin and increased Tbet^hi^Eomes^dim^ phenotype, compared to Vδ1^+^ γδT-cells from CHB and NC subjects. In this regard, the poor effector function of Vδ2^+^ γδT-cells from AHB subjects may reflect their enrichment for Tbet^dim^Eomes^hi^ phenotype associated with exhausted HIV-specific CD8 T-cells with increased PD1 expression [65]. However, alternative mechanisms are likely, as PD1 expression was not increased in Vδ2^+^ γδT-cells from AHB subjects and did not correlate with IFNγ/TNF expression, despite positive correlations between PD1 expression and Tbet^dim^Eomes^hi^ phenotype in Vδ2^+^ γδT-cells. Furthermore, we cannot rule out hepatic compartmentalization of more functional γδT-cells in AHB. Third, late IFNγ/TNF responses to pAg in Vδ2^+^ γδT-cells correlated with their expression of NKG2A/CD94 and CD158a as well as Tbet^hi^Eomes^dim^ phenotype. For PMA/Ionomycin, late responses in Vδ2^+^ γδT-cells correlated significantly with CD94 and Tbet/Eomes expression, whereas early responses correlated with NKG2A and CD94 but not Tbet/Eomes expression. In this regard, increased NKG2A and CD94 expression has been associated with more cytolytic Vδ2^+^ γδT-cells [83] although direct stimulation through CD94/NKG2A is inhibitory to Vδ2^+^ γδT-cells [23, 84]. Collectively, these findings show that circulating Vδ2^+^ γδT-cells are functionally preserved in CHB but suppressed in AHB, with regulatory roles for NK inhibitory receptors and Tbet/Eomes in their effector function, without increased T-cell activation or regulatory markers.

Surprisingly, early IFNγ/TNF responses in Vδ2^+^ γδT-cells to PMA/Ionomycin stimulation showed significant inverse correlations with serum ALT (but not HBV DNA) levels, despite non-specific nature of PMA/Ionomycin stimulation that bypasses the early steps of TCR signaling [69, 80]. Moreover, Vδ2^+^ γδT-cells showed less IFNγ/TNF expression and more “anergic” IFNγ^-^TNF^-^ double-negative phenotype in CHB Flare compared to CHB Non-flare subjects. Conversely, CHB Non-flare subjects showed greater IFNγ/TNF expression in Vδ2^+^ γδT-cells compared to uninfected controls and AHB subjects. Significant inverse association was also detected for early IFNγ responses to PMA/Ionomycin in Vδ1^+^ γδT-cells, although limited to IFNγ and without significant differences between CHB with and without ALT flare. However, serum ALT did not correlate with pAg-specific IFNγ/TNF responses in Vδ2^+^ γδT-cells, although pAgs provide more specific and physiological stimulation to Vδ2^+^ γδT-cells [16, 25–28]. Collectively, these findings suggest that circulating γδT-cells are functionally altered in CHB and that their early IFNγ/TNF responses to PMA/Ionomycin may provide a biomarker for immune active phenotype beyond ALT.

Given the known antiviral effects of IFNγ and TNF against HBV [4–9], it was surprising that HBV DNA levels in CHB did not correlate with the circulating frequency or IFNγ/TNF expression by γδT-cells. This lack of correlation could reflect insufficient in-vivo activation of γδT-cells to exert antiviral activity (especially given increased expression of inhibitory NKG2A/CD94 receptors) although Vδ2^+^ γδT-cells from our CHB subjects were responsive to phosphoantigens in-vitro. In fact, circulating γδT-cells from CHB, NC and AHB subjects showed little to no cytokine expression when stained without further stimulation in-vitro (**Figs 4B, 6A and 6B**). As a caveat, it should be acknowledged that we did not study the intrahepatic compartment which is enriched in γδT-cells, although both Vδ1^+^ and Vδ2^+^ γδT-cells have been detected in the liver of HBV-infected and uninfected subjects [45, 85].

The inverse association between Vδ2^+^ γδT-cell function and ALT activity might also suggest a protective role for Vδ2^+^ γδT-cells against HBV-associated liver inflammation and hepatocellular injury. In fact, a late tissue protective and immune regulatory role have been suggested for γδT-cells, by directly killing activated macrophages, producing regulatory cytokines and secreting factors that promote tissue repair, beyond early pro-inflammatory effects [15, 86–90]. Consistent with this possibility, Vδ2^+^ γδT-cell depletion and anergy have been associated with adverse outcomes in HIV-infection [37–39]. Although beyond the scope of this study, potential regulatory roles in HBV pathogenesis have been suggested for γδT-cells with IFNγ-dependent suppression of Th17^+^ CD4 T-cells [45] and the induction of myeloid-derived suppressor cells [49]. In this context, it is tempting to speculate if hepatitis flare in CHB might represent a failure of IFNγ−dependent regulatory function by Vδ2^+^ γδT-cells.

Alternatively, IFNγ/TNF deficit in Vδ2^+^ γδT-cells from CHB flare could be a consequence of inflammatory and regulatory mediators induced during active hepatocellular injury in-vivo [12, 91, 92], as CHB flares are associated with necroinflammatory changes in the liver [71–73]. However, IFNγ/TNF expression by Vδ2^+^ γδT-cells did not improve even months after the resolution of CHB flare (including in one subject with therapeutic control of HBV DNA). In this regard, persistent IFNγ/TNF deficit in Vδ2^+^ γδT-cells from CHB Flare patients may represent continued cellular stress with fibrogenesis and cell turnover in the chronicallly HBV-infected liver despite apparent improvement in ALT, although we could not address this question due to limited number of liver biopsy in our study. By contrast, early IFNγ response to brief PMA/Ionomycin stimulation improved in Vδ2^+^ γδT-cells from AHB subjects after 2–7 months from initial presentation, suggesting that regulatory effect on Vδ2^+^ γδT-cell function is reversible in AHB unlike CHB.

In conclusion, circulating γδT-cells are preserved in frequency and function, but with distinct and innate phenotype in acute and chronic HBV infection, with a significant inverse associations between early IFNγ/TNF responses in Vδ2^+^ γδT-cells and serum ALT in CHB. Our findings suggest that circulating γδT-cells participate in lymphoid stress surveillance in HBV infection, with differential activation and differentiation with potential relevance to HBV pathogenesis.

## Supporting information

S1 Fig Comparisons of cytokine responses in Vδ2^+^ γδT-cells following 8 and 23 hours in-vitro stimulation. **A.** Histogram overlay of IFNγ/TNF responses in Vδ2^+^ γδT-cells following in-vitro stimulation for 8h (gray shaded) versus 23 hours (red line) with media control, phosphoantigens zoledronic acid (Zol), (E)-4-hydroxy-3-methyl-but-2-enyl pyrophosphate (HMBPP) and PMA/Ionomycin in-vitro as described in Methods. **B**. Dot plots show quadrant analysis for IFNγ and/or TNF expression in Vδ2^+^ γδT-cells following Zol or PMA/Ionomycin stimulation. As shown, IFNγ/TNF responses in Vδ2^+^ γδT-cells to pAg were greater with longer 23 hours of stimulation compared to 8 hours. For P/I, IFNγ/TNF responses in Vδ2^+^ γδT-cells were greater with shorter 8 hours of stimulation compared to 23 hours.
(TIF)

S2 Fig Patterns of Tbet/Eomes expression and/or IFNγ/TNF phenotype by Vδ2^+^ γδT-cells relative to race/ethnicity and age. **A.** Bar graphs comparing median %Tbet^+^, %Eomes^+^, %Tbet^hi^ Eomes^dim^, %Tbet^dim^ Eomes^hi^ in Vδ2^+^ γδT-cells between 29 CHB and 12 NC subjects among Asians (top panel) and between 7 CHB, 12 NC and 7 AHB subjects among Non-Asians (bottom panel). Among Asians, CHB was associated with significantly greater %Eomes/Vδ2^+^ γδT-cells compared to NC (p = .0007) by Mann Whitney U. Among Non-Asians, AHB was associated with significantly lower %Tbet^+^ (p = .04) but greater %Tbet^dim^ Eomes^hi^ (p = .01) in Vδ2^+^ γδT-cells compared to CHB and NC subjects by Kruskal Wallis (k = 3). **B.** Scatter plots comparing age with %Tbet^+^, %Eomes^+^, %Tbet^hi^ Eomes^dim^, %Tbet^dim^ Eomes^hi^ in Vδ2^+^ γδT-cells without significant correlations by non-parametric Spearman rank order correlations. **C.** Bar graphs comparing median %IFNγ^+^, %TNF^+^, %IFNγ^+^ TNF^+^ in Vδ2^+^ γδT-cells between 29 CHB and 12 NC subjects among Asians by Mann Whitney U (top panel) and between 7 CHB, 12 NC and 7 AHB subjects among Non-Asians by Kruskal Wallis (k = 3) (bottom panel). **D.** Scatter plots comparing age with %IFNγ^+^, %TNF^+^, %IFNγ^+^ TNF^+^ in Vδ2^+^ γδT-cells without significant correlations by non-parametric Spearman rank order correlations. P-values < 0.05 were considered statistically significant.
(TIF)

S3 Fig Correlations between γδT-cell expression of NK/T-cell markers, relative to their Tbet/Eomes expression and clinical parameters. **A.** Scatter plots compare %Tbet^+^, %Eomes^+^, %Tbet^hi^ Eomes^dim^, %Tbet^dim^ Eomes^hi^ in Vδ1^+^ γδT-cells to their %CD56, %CD16 and %CD161. **B.** Scatter plots compare expression of NK/T-cell markers in CD3^hi^CD4^-^ T-cells with serum HBV DNA and ALT. Correlation coefficients and p-values were calculated by Spearman rank order correlation. Significantly positive correlations are shown in red font whereas significantly negative correlations are shown in blue font, with p-values <0.05 considered significant.
(TIF)

S4 Fig Gating strategy to examine IFNγ, TNF and/or MIP1β co-expression in circulating CD3^hi^CD4- γδT-cells. CD3^hi^CD4^-^ γδT-cells are gated and examined for IFNγ and/or TNF expression by quadrant gating, followed by histogram analysis for presence or absence of MIPβ1 expression.
(TIF)

S5 Fig Expression of IFNγ and TNF but not IL17 upon pAg stimulation in Vδ2^+^ γδT-cells in PBMC. Cytokine expression in Vδ2^+^ γδT-cells (gated by Vγ9 TCR expression as shown on the far left FACS file) following 23 hours of culture with media control, Zol, HMBPP and PMA/Ionomycin is shown in pseudocolor plots, with IFNγ and TNF but not IL17 expression in response to pAg and PMA/Ionomycin.
(TIF)

S6 Fig Correlations between IFNγ/TNF responses to various stimulation conditions. **A**. Scatter plots comparing %IFNγ^+^, %IFNγ^+^TNF^+^ and %TNF^+^ between Vδ2^+^ γδT-cells stimulated for 23 hours with zoledronic acid (Zol), (E)-4-hydroxy-3-methylbut-2-enyl 4-diphosphate (HMBPP) or PMA/Ionomycin (P/I). **B**. Scatter plots comparing %IFNγ^+^, %IFNγ^+^TNF^+^ and %TNF^+^ in Vδ2^+^ γδT-cells stimulated for 23 hours with PMA/Ionomycin (P/I), zoledronic acid (Zol) and (E)-4-hydroxy-3-methylbut-2-enyl 4-diphosphate (HMBPP) in Vδ2^+^ γδT-cells on the y-axis, with same parameters following 5 hours of stimulation with P/I on the x-axis. Correlation coefficients and p-values calculated by Spearman rank order correlation. For convenience, significantly positive correlations are shown in red font, with p-values <0.05 considered significant.
(TIF)
