# Supplementary figures and images for: Distinct phenotype and function of circulating Vδ1+ and Vδ2+ γδT-cells in acute and chronic hepatitis B
Source: PLoS Pathog. 2019 Apr 18;15(4):e1007715. doi: 10.1371/journal.ppat.1007715 (PMC6490945; doi:10.1371/journal.ppat.1007715)

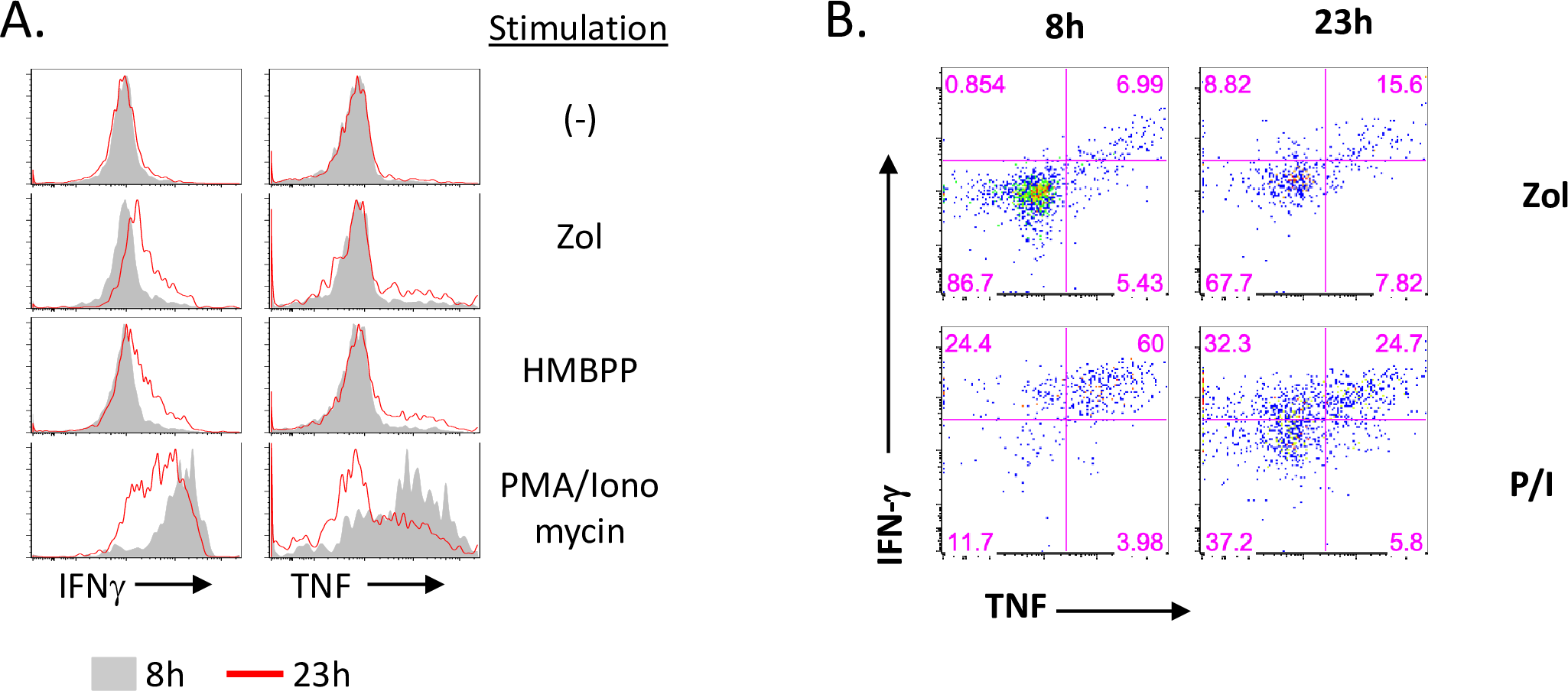

Supplement: S1 Fig — A. Histogram overlay of IFNγ/TNF responses in Vδ2+ γδT-cells following in-vitro stimulation for 8h (gray shaded) versus 23 hours (red line) with media control, phosphoantigens zoledronic acid (Zol), (E)-4-hydroxy-3-methyl-but-2-enyl pyrophosphate (HMBPP) and PMA/Ionomycin in-vitro as described in Methods. B. Dot plots show quadrant analysis for IFNγ and/or TNF expression in Vδ2+ γδT-cells following Zol or PMA/Ionomycin stimulation. As shown, IFNγ/TNF responses in Vδ2+ γδT-cells to pAg were greater with longer 23 hours of stimulation compared to 8 hours. For P/I, IFNγ/TNF responses in Vδ2+ γδT-cells were greater with shorter 8 hours of stimulation compared to 23 hours. (TIF) [file ppat.1007715.s001.tif]

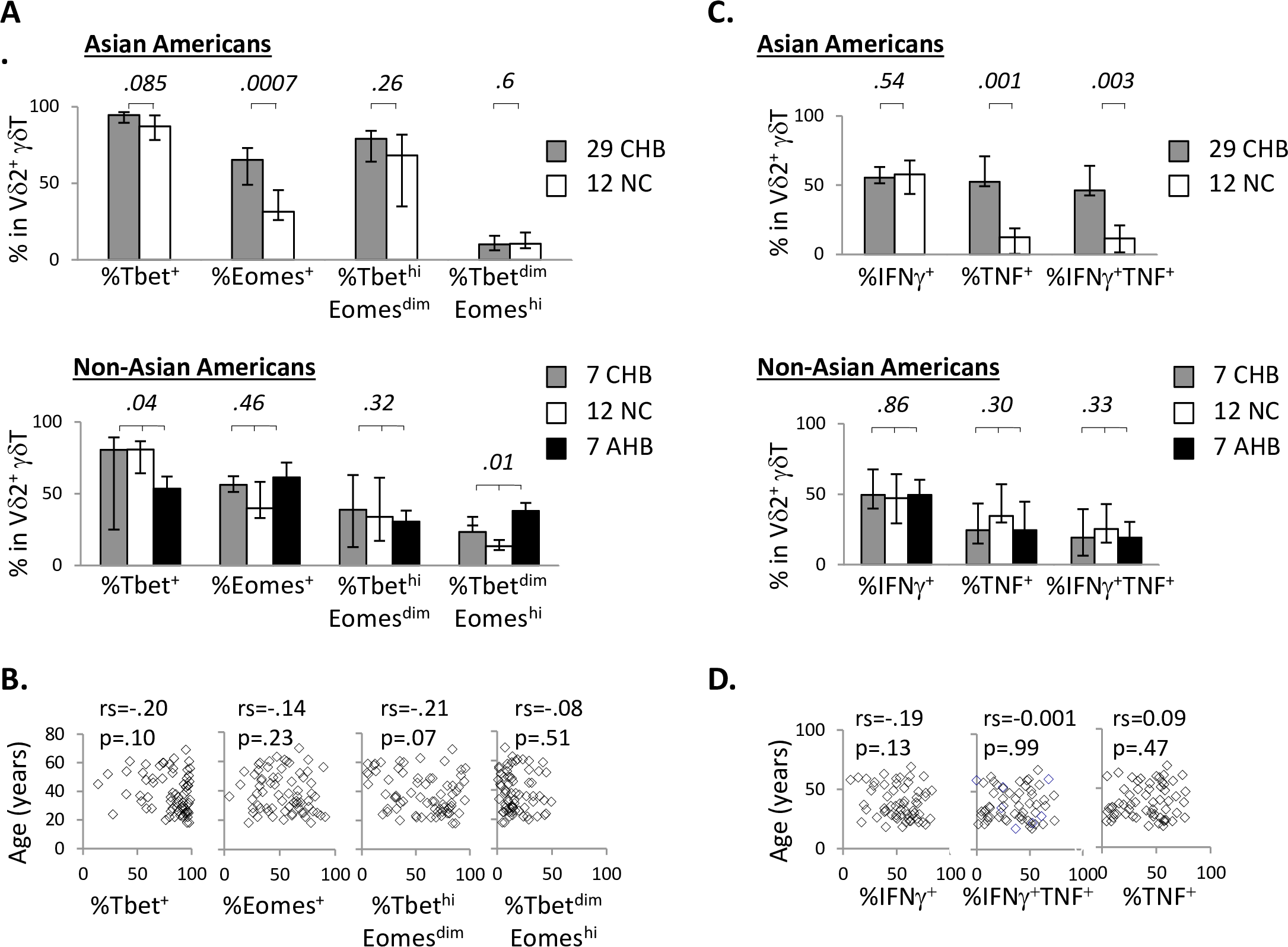

Supplement: S2 Fig — A. Bar graphs comparing median %Tbet+, %Eomes+, %Tbethi Eomesdim, %Tbetdim Eomeshi in Vδ2+ γδT-cells between 29 CHB and 12 NC subjects among Asians (top panel) and between 7 CHB, 12 NC and 7 AHB subjects among Non-Asians (bottom panel). Among Asians, CHB was associated with significantly greater %Eomes/Vδ2+ γδT-cells compared to NC (p = .0007) by Mann Whitney U. Among Non-Asians, AHB was associated with significantly lower %Tbet+ (p = .04) but greater %Tbetdim Eomeshi (p = .01) in Vδ2+ γδT-cells compared to CHB and NC subjects by Kruskal Wallis (k = 3). B. Scatter plots comparing age with %Tbet+, %Eomes+, %Tbethi Eomesdim, %Tbetdim Eomeshi in Vδ2+ γδT-cells without significant correlations by non-parametric Spearman rank order correlations. C. Bar graphs comparing median %IFNγ+, %TNF+, %IFNγ+ TNF+ in Vδ2+ γδT-cells between 29 CHB and 12 NC subjects among Asians by Mann Whitney U (top panel) and between 7 CHB, 12 NC and 7 AHB subjects among Non-Asians by Kruskal Wallis (k = 3) (bottom panel). D. Scatter plots comparing age with %IFNγ+, %TNF+, %IFNγ+ TNF+ in Vδ2+ γδT-cells without significant correlations by non-parametric Spearman rank order correlations. P-values < 0.05 were considered statistically significant. (TIF) [file ppat.1007715.s002.tif]

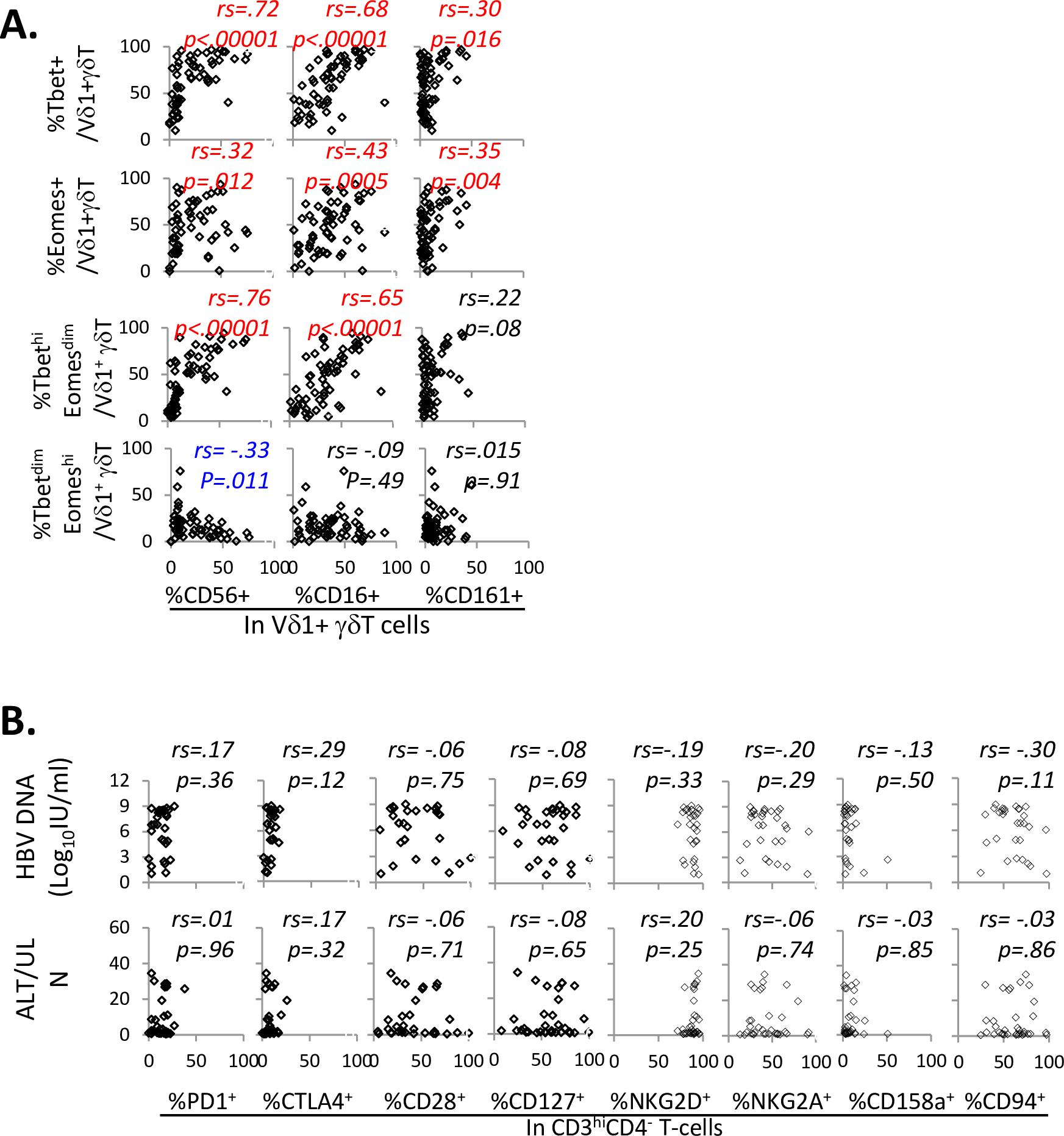

Supplement: S3 Fig — A. Scatter plots compare %Tbet+, %Eomes+, %Tbethi Eomesdim, %Tbetdim Eomeshi in Vδ1+ γδT-cells to their %CD56, %CD16 and %CD161. B. Scatter plots compare expression of NK/T-cell markers in CD3hiCD4- T-cells with serum HBV DNA and ALT. Correlation coefficients and p-values were calculated by Spearman rank order correlation. Significantly positive correlations are shown in red font whereas significantly negative correlations are shown in blue font, with p-values <0.05 considered significant. (TIF) [file ppat.1007715.s003.tif]

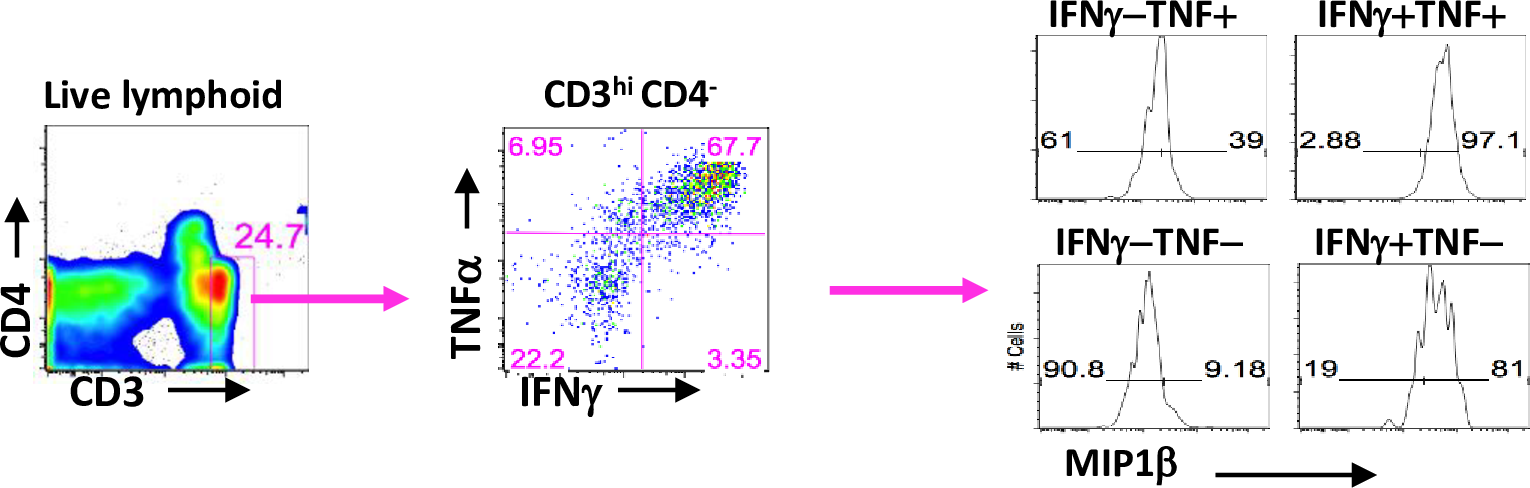

Supplement: S4 Fig — CD3hiCD4- γδT-cells are gated and examined for IFNγ and/or TNF expression by quadrant gating, followed by histogram analysis for presence or absence of MIPβ1 expression. (TIF) [file ppat.1007715.s004.tif]

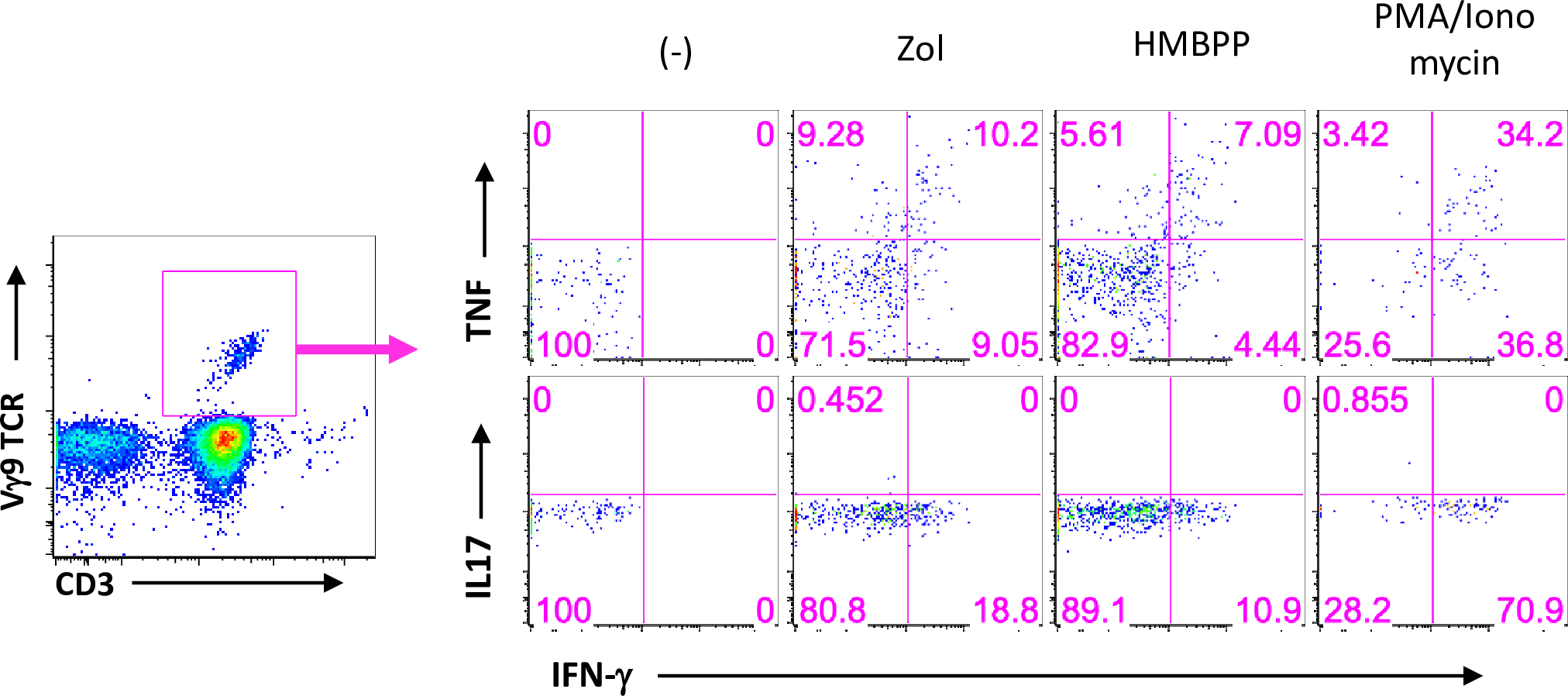

Supplement: S5 Fig — Cytokine expression in Vδ2+ γδT-cells (gated by Vγ9 TCR expression as shown on the far left FACS file) following 23 hours of culture with media control, Zol, HMBPP and PMA/Ionomycin is shown in pseudocolor plots, with IFNγ and TNF but not IL17 expression in response to pAg and PMA/Ionomycin. (TIF) [file ppat.1007715.s005.tif]

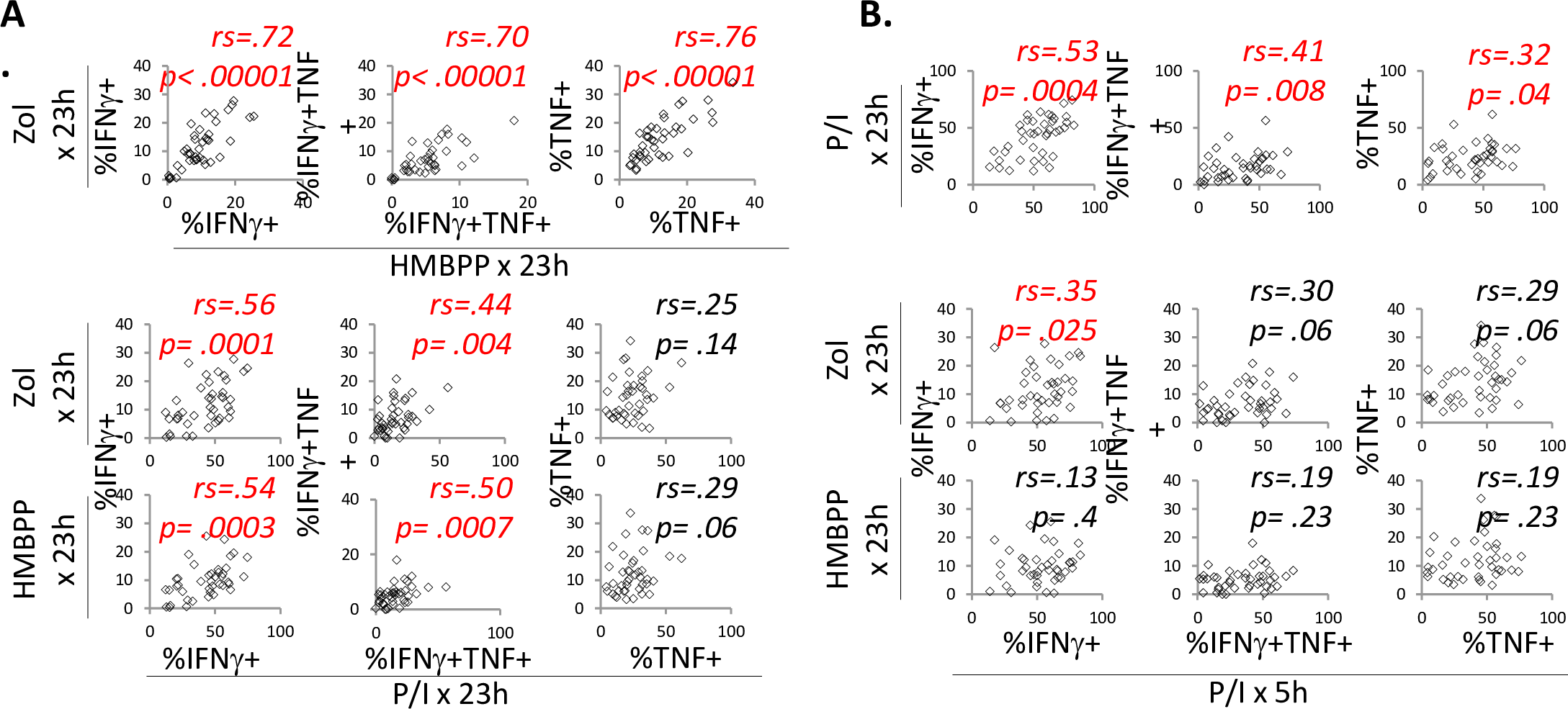

Supplement: S6 Fig — A. Scatter plots comparing %IFNγ+, %IFNγ+TNF+ and %TNF+ between Vδ2+ γδT-cells stimulated for 23 hours with zoledronic acid (Zol), (E)-4-hydroxy-3-methylbut-2-enyl 4-diphosphate (HMBPP) or PMA/Ionomycin (P/I). B. Scatter plots comparing %IFNγ+, %IFNγ+TNF+ and %TNF+ in Vδ2+ γδT-cells stimulated for 23 hours with PMA/Ionomycin (P/I), zoledronic acid (Zol) and (E)-4-hydroxy-3-methylbut-2-enyl 4-diphosphate (HMBPP) in Vδ2+ γδT-cells on the y-axis, with same parameters following 5 hours of stimulation with P/I on the x-axis. Correlation coefficients and p-values calculated by Spearman rank order correlation. For convenience, significantly positive correlations are shown in red font, with p-values <0.05 considered significant. (TIF) [file ppat.1007715.s006.tif]
